# NuSeT: A deep learning tool for reliably separating and analyzing crowded cells

**DOI:** 10.1371/journal.pcbi.1008193

**Published:** 2020-09-14

**Authors:** Linfeng Yang, Rajarshi P. Ghosh, J. Matthew Franklin, Simon Chen, Chenyu You, Raja R. Narayan, Marc L. Melcher, Jan T. Liphardt

**Affiliations:** 1 Bioengineering, Stanford University, Stanford, CA, United States of America; 2 BioX Institute, Stanford University, Stanford, CA, United States of America; 3 ChEM-H, Stanford University, Stanford, CA, United States of America; 4 Cell Biology Division, Stanford Cancer Institute, Stanford, CA, United States of America; 5 Chemical Engineering, Stanford University, Stanford, CA, United States of America; 6 Department of Pathology, Stanford University School of Medicine, Stanford, CA, United States of America; 7 Electrical Engineering, Stanford University, Stanford, CA, United States of America; 8 Department of Surgery, Stanford University School of Medicine, Stanford, CA, United States of America; Northeastern University, UNITED STATES

## Abstract

Segmenting cell nuclei within microscopy images is a ubiquitous task in biological research and clinical applications. Unfortunately, segmenting low-contrast overlapping objects that may be tightly packed is a major bottleneck in standard deep learning-based models. We report a Nuclear Segmentation Tool (NuSeT) based on deep learning that accurately segments nuclei across multiple types of fluorescence imaging data. Using a hybrid network consisting of U-Net and Region Proposal Networks (RPN), followed by a watershed step, we have achieved superior performance in detecting and delineating nuclear boundaries in 2D and 3D images of varying complexities. By using foreground normalization and additional training on synthetic images containing non-cellular artifacts, NuSeT improves nuclear detection and reduces false positives. NuSeT addresses common challenges in nuclear segmentation such as variability in nuclear signal and shape, limited training sample size, and sample preparation artifacts. Compared to other segmentation models, NuSeT consistently fares better in generating accurate segmentation masks and assigning boundaries for touching nuclei.

## Author summary

Nuclear size and shape are essential indicators of cell cycle stage and cellular pathology. Efficient segmentation of nuclei in complex environments, especially for high-value yet low-quality samples is critical for detecting pathological states. In the majority of cases, biological features are still segmented using traditional segmentation methods requiring manual curation of segmentations, which is hugely time-consuming and does not achieve optimal performance. While a recent surge in deep learning tools has helped tremendously with the automation of segmentation tasks, existing platforms inefficiently segment nuclei in crowded cells with overlapping nuclear boundaries. NuSeT, assimilates the advantages of semantic segmentation (U-Net) and instance segmentation (Mask R-CNN), and consistently outperforms other start-of-the-art deep learning segmentation models in analyzing complex three-dimensional cell clusters and in tracking nuclei in crowded, dynamic environments. NuSeT can work with both fluorescent and histopathology image samples. We have also developed a graphic user interface for customized training and segmentation, that will aid considerably in the ease and accuracy of image segmentation in a wide range of image types.

This is a *PLOS Computational Biology* Methods paper

## Introduction

Quantitative single-cell analysis can reveal novel molecular details of cellular processes relevant to basic research, drug discovery, and clinical diagnostics. For example, cell morphology and shape are reliable proxies for cellular health and cell-cycle stage, as well as indicating the state of disease-relevant cellular behaviors such as adhesion, contractility, and mobility.[1–5] However, accurate segmentation of cellular features such as the size and shape of the nucleus remains challenging due to large variability in signal intensity and shape, and artifacts introduced during sample preparation.[6,7] These challenges are exacerbated by cellular crowding, which juxtaposes cells and obscures their boundaries. Additionally, in many traditional segmentation methods[8], parameters need to be iteratively adjusted for images varying in quality.[9]

Convolutional neural networks (CNN) have emerged as a robust alternative to traditional segmentation methods for segmenting cell nuclei.[10–16] CNNs achieve their superior performance through new deep-learning models.[10,17–19] CNNs’ applicability for high precision image segmentation was first demonstrated by a Fully Convolutional Network (FCN) for pixel-level segmentation.[10] Additional FCN cell segmentation models have since been developed.[14,20,21] These pioneering approaches established a basic pipeline for CNN-based nuclear segmentation and achieved significant improvements in segmenting different types of cells including bacteria and mammalian cells.[14,21] However, in their original form, FCNs typically required large training datasets to achieve high levels of accuracy.[10] This bottleneck was overcome in U-Net by introducing a U-shaped network that incorporates pooling layers and up-sampling layers.[15] Additionally in U-Net, the network was guided to segment overlapping objects by introducing weight matrices at cell-boundaries. Several state-of-the-art nuclear segmentation models have since been developed using this architecture.[11,13,22,23] Several online cell segmentation interfaces allow users to predict and train on their own image data, facilitating front end use by researchers.[23,24] However, U-Net and FCN based models are curated and evaluated on pixel-level accuracy, where each pixel is segmented directly without the object detection step. In cell biology, the main goal is to make reliable statements about *cells* as a whole (e.g. the number of cells, their average size and shape, detection of rare/unusual cells) rather than focusing on image pixels. For such problems, the idea of instance segmentation provides a more effective solution, as the loss function incorporates a sense of the whole object and not just individual pixels. One such approach, the Deep watershed transform[25], incorporates the object by learning a distance transform computed from the original training masks. The distance transform is further fed into a watershed layer to have the final segmentation results. A recent improvement is to incorporate a Faster R-CNN detection module. In this approach, the algorithm computes object locations and uses them as markers for the watershed layer, improving the segmentation.[26] Another approach, Mask R-CNN[19], applies FCN-based segmentation to regions proposed by Region Proposal Networks (RPN) and achieves good segmentation results in real-world image datasets. A more recent implementation of this approach replaces the RPN with a single shot detection module[27], achieving superior performance in segmenting and tracking cells and nuclei.[28,29] However, the performance of Mask R-CNN based approaches remains to be validated for images with high cell density. Mask R-CNN also employs fixed anchor scales for bounding boxes across all images, which is a limitation for samples with variable-sized nuclei.[18,19] Additionally, at the pixel-level, the segmentation task of Mask R-CNN is performed by FCN, which is less accurate with small training datasets compared with U-Net.[15,30]

To address these issues, we have developed a Nuclei Segmentation Toolset (NuSeT), which integrates U-Net[15] and a modified RPN (based on the implementation of previous works [31,32]) to accurately segment fluorescently labeled nuclei. In this integrated model, U-Net performs pixel-segmentation, while the modified RPN predicts unique bounding boxes for each image based on U-Net segmentations. The resulting output provides seeds for a watershed algorithm to segment touching nuclei. To minimize segmentation errors stemming from fluorescence signal variability and cell density variability in samples, we employed a novel normalization method that uses only foreground pixel intensities for image normalization. To increase the robustness and applicability of the model, we used training sets including samples with wide variations in imaging conditions, image dimensions, and non-cellular artifacts. Extensive qualitative and quantitative evaluation suggest that our segmentation pipeline significantly improves nuclei segmentation, especially in distinguishing overlapping boundaries, and is generalizable to both fluorescent and histopathological images.

## Results

### NuSeT is a robust nuclear segmentation tool

Tools for segmenting fluorescent nuclei need to address multiple features and limitations of biological images.[6,33] Typical issues and limitations include:

1. Boundary assignment ambiguity: biological samples frequently have very high cell density with significant overlap between objects.
2. Signal intensity variation: Within one image, the signal can vary within each nucleus (e.g. due to different compaction states of the DNA in heterochromatin vs. euchromatin) and across nuclei (e.g. due to cell to cell differences in nuclear protein expression levels and differences in staining efficiency).
3. Non-cellular artifacts and contaminants: Fluorescence microscopy samples are often contaminated with auto-fluorescent cell debris as well as non-cellular artifacts.
4. Low signal to noise ratios (SNRs): Low SNRs typically result from lower expression levels of fluorescent targets and/or high background signal, such as sample autofluorescence. (S1 Fig).

**Fig 1:**
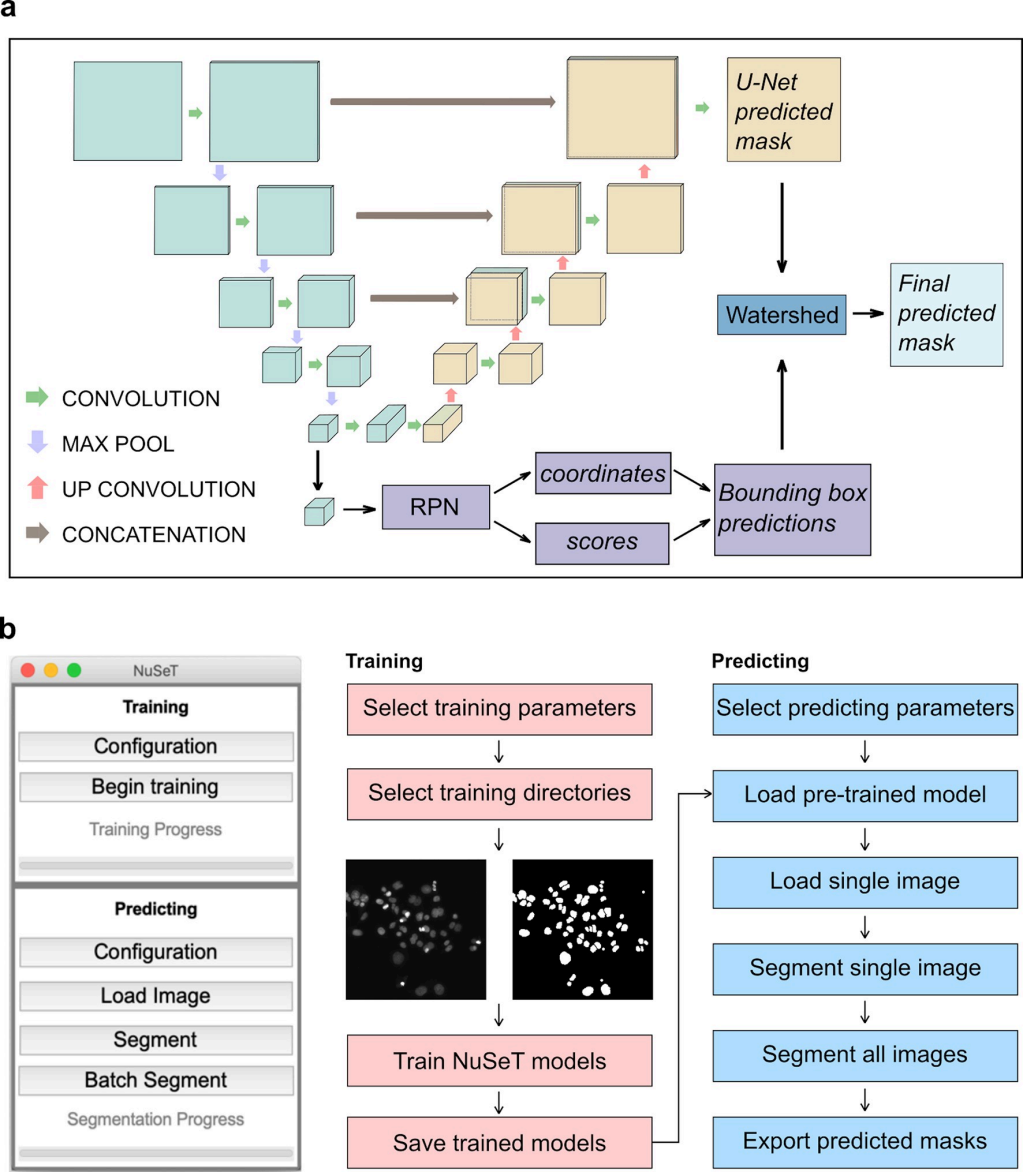
Pipeline for segmenting nuclei with NuSeT. (**A**) Deep-learning model structure of NuSeT. The inputs of the model are gray scale images with different sizes. The outputs are binary masks with the same size as inputs, with predicted foreground regions as Ones and background regions as Zeroes. The model combines U-Net (gray and orange) and Region Proposal Network (purple), which performs nuclei segmentation and detection separately. The results are then merged and processed by watershed (dark blue) to generate final predictions. (**B**) Outlook of NuSeT Graphic User Interface(GUI), and example training and predicting pipelines using NuSeT GUI.

We used an end-to-end training approach that incorporates both U-Net and Region Proposal Network (RPN)[15,18] to address these issues (Methods). In our approach, the training and inference step consists of running an input image in parallel in both U-Net and RPN. The final output of U-Net consists of two feature maps of the same shape as the input image, representing background and foreground pixel-assignment scores.[10] The final foreground prediction is then computed from the maximum class score of each pixel. Although U-Net alone performs well on some microscopy datasets[30,34], we incorporated RPN since it was originally designed to detect objects in images with high information content.[18] We reasoned that the accurate performance of RPN in detecting objects can be leveraged to improve nuclear segmentation performance. To achieve robust separation of touching nuclei, we used RPN bounding boxes to determine nuclear centroids, which were then supplied as seeds to a watershed algorithm.[35,36] To improve segmentation accuracy in images with large nuclear size variations, we modified the original RPN architecture to use bounding box dimensions based on average nuclear size for each image (S2 Fig). Instead of training U-Net and RPN separately, we merged the feature-extraction part of RPN with the down-sampling part of U-Net to avoid longer training time and more memory cost (Fig 1A).[10,15,18,19] In this way, the instance detection insights of RPN are extracted from the model structure. To evaluate the segmentation performance of the different algorithms, we computed the mean intersection over union for foreground and background (mean IoU), Root Mean Square Error (RMSE), and pixel accuracy (to benchmark pixel-level performance). Since in biological image processing the primary focus is on cell-level segmentation rather than pixel-level accuracy, we also included object-level segmentation metrics, including the rate of correctly separating overlapping nuclei, correct and incorrect detections, splits, merges, catastrophes, and both the false-positive and false-negative detection rates (Methods).[29,30] Two separate datasets, ‘MCF10A’ and ‘Kaggle’, were used to compare the performance of the algorithms.[33] The MCF10A dataset consists of images of relatively uniformly fluorescent nuclei of a non-tumorigenic breast epithelial cell line[37], grown to different levels of confluence. The Kaggle dataset was adapted from a public dataset[33] representing cells from different organisms (including humans, mice, and flies) and containing images with a wide range of brightness, cell densities, and nuclear sizes. The overall comparison in S1 Table and S2 Table suggests that NuSeT achieves similar pixel-level segmentation accuracy compared with a current state-of-the-art pixel-level cell segmentation approach (U-Net) but has higher separation rates for overlapping nuclei and fewer merge errors. With the Kaggle dataset, NuSeT improved the separation of touching nuclei by more than 75% compared with U-Net. Compared with another state-of-the-art instance segmentation approach, Mask R-CNN, NuSeT achieved much lower false-negative detection rates in Kaggle dataset, leading to significantly better pixel-level segmentation accuracy. To make NuSeT more user-friendly, we have prepared a cross-platform graphic user interface (GUI) for the scientific community. Our GUI comes with the pretrained model which we used to benchmark NuSeT performance for various nuclei segmentation tasks. The GUI also allows the use of training and predicting modules (Fig 1B), allowing the users to perform custom segmentation tasks with NuSeT.

### Foreground normalization improves segmentation performance

**Fig 2:**
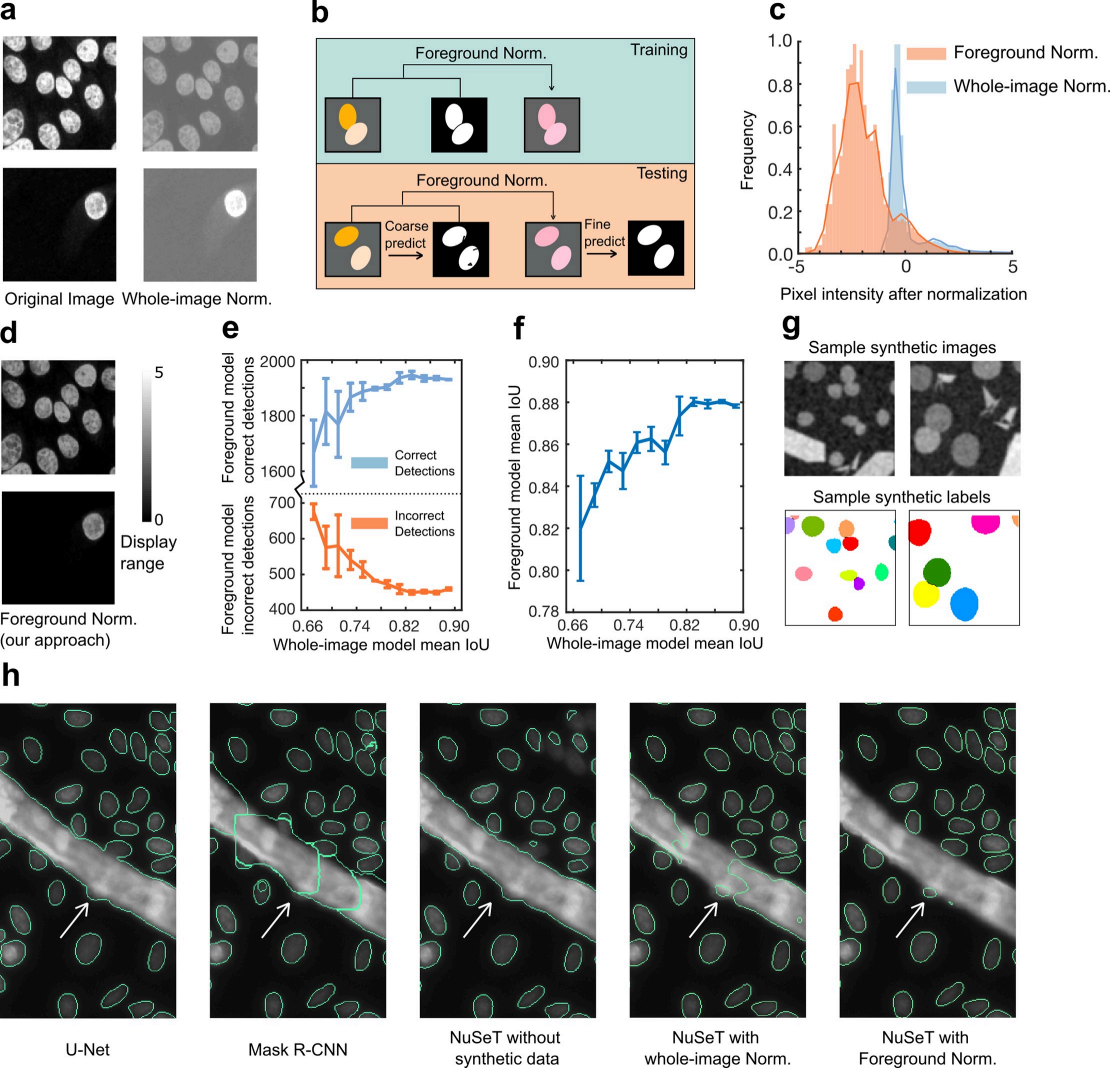
Improved normalization performance by foreground normalization and synthetic training. **(A)** The visual effects of normalizing sparse/dense samples using whole-image normalization showing images having inconsistent nuclear signals after normalization. **(B)** Foreground normalization during training and testing. During training, only pixels belonging to cell nuclei are used to normalize the image. During testing, a coarse segmentation prediction is generated by the model, and pixels belonging to the predicted nuclei are used to perform foreground normalization. The model then makes final predictions based on the normalized input images. **(C)** Distribution of pixel intensities over an entire training dataset after different normalizations, showing foreground normalization has wider dynamic range. **(D)** The visual effects of normalizing sparse/dense samples using foreground normalization showing images have a higher dynamic range and more consistent nuclear signals. **(E, F)** Line charts showing that the object-level performance **(E)** and the pixel-level performance **(F)** of the foreground normalization model depend on the pixel-level performance of the whole-image normalization model. Error bars represent three individual experiments. **(G)** Examples of synthetic images with labels used during training. Our algorithm can generate synthetic nuclei-shaped blobs with different sizes, as well as different types of artifacts to increase the robustness of the model. Overlapping nuclei were introduced to enhance NuSeT performance in touching nuclei separation. **(H)** Representative examples comparing the performances of different segmentation approaches. Training without synthetic images mis-identified artifacts (stripes) as foreground. The addition of synthetic data improved artifact detection. Switching to foreground normalization led the best performance including robust identification of imaging artifact, detected of more nuclei, and better separation of touching nuclei compared to Mask R-CNN and U-Net.

Normalizing training data to alleviate image intensity differences is central to accelerating learning and improving network performance. Historically, imaging data have been normalized by subtracting the mean intensity calculated from all pixels in a dataset.[38,39] However, this leads to discrepancies in normalization, particularly for images with markedly different brightness levels. Normalizing data at whole-image level addresses the issue of illumination differences[40], but introduces brightness differences in images with sub-regions of varying cell densities (Fig 2A). Additionally, whole-image normalization fares poorly in images strewn with auto-fluorescent artifacts (S3 Fig). We incorporated a foreground normalization step in our data preprocessing. In this approach, only the pixels that belong to cell nuclei (foreground) are selected to calculate mean and standard deviation of pixel intensities. Since no label is provided during inference, foreground normalization requires two passes. In step one, the test data are normalized on a per image level to generate a coarse prediction of the foreground with our RPN-U-Net fusion. In step two, this coarse prediction is used to perform foreground normalization on test images before they are fed into the model for a second pass (Fig 2B). Compared with whole-image normalization, the two-step foreground normalization approach is relatively robust to illumination differences, cell-density variations, and image artifacts and performs better in normalizing images with a broader dynamic range of pixel intensities (Fig 2C and 2D). As a result, model training with foreground normalization increased nuclei detection accuracy and boundary assignment for both Kaggle and MCF10A datasets, with more correct detections and less merge errors (S1 Table).

To further analyze how whole-image normalization models affect the performance of the foreground normalization model, we trained the whole-image normalization models to different mean IoU levels. This step was essential as the pixel-level accuracy of the whole-image normalization model was critical for selecting pixels to perform the following foreground normalization. By connecting the different whole-image normalization models with the final foreground normalization model, we found that when the mean IoU of the whole-image normalization models were less than 0.82, the performance of the foreground normalization model heavily depended on the whole-image normalization models (Fig 2E and 2F, S3 Table). This suggests that the performance of the foreground normalization model relies on the accuracy level of the whole-image normalization model. However, when the mean IoU of the whole-image normalization models were higher than 0.82, the foreground normalization model was less affected (Fig 2E and 2F, S3 Table).

Given the modularity of the foreground normalization approach, we next asked whether foreground normalization could be integrated into other deep learning models, such as U-Net and Mask R-CNN, to enhance their performance. Consistent with our expectation, training U-Net with foreground normalization improved the overlapping nuclei separation performance by 6% to 35% (in MCF10A and Kaggle datasets). Foreground normalization also improved nuclei-detection accuracy of U-Net, and reduced merge errors (S4 Table). However, the segmentation performance of Mask R-CNN was not significantly improved by foreground normalization. The segmentation performance was almost identical to the model trained with whole-image normalization (S4 Table). Given that the performance of Mask R-CNN is highly dependent on the detection accuracy of RPN, whereas both NuSeT and U-Net rely heavily on pixel classification to perform segmentation, we concluded that foreground normalization improved the segmentation performance by rescaling the image pixels more consistently, aiding in better classification of foreground and background pixels.

### Synthetic datasets in model-training improve detection and segmentation accuracy

Common sample contaminants have irregular shapes, significantly different overall brightness levels and aspect ratios compared to real cells, and uneven pixel intensities. To improve model performance and minimize false-positive detection rates, we computationally generated synthetic images containing irregular shapes with varying intensities, as well as nuclei-like blobs (Methods). We also added Gaussian blur and noise to the synthetic images to better represent real-world images. Additionally, overlapping blobs were included to mimic touching nuclei. Example synthetic images and training labels are shown in Fig 2E. Including synthetic data in the training process notably improved the model’s performance in distinguishing real nuclei from imaging artifacts (Fig 2F) and enhanced the separation of touching nuclei (S1 Table). The addition of foreground normalization on top of the synthetic images during model-training further reduced false positive detections (Fig 2F). Aided by these improvements, NuSeT outperformed both U-Net and Mask R-CNN in artifact detection/rejection (Fig 2F).

### RPN-aided Watershed improves boundary-resolution of highly overlapping objects

Having improved nuclear segmentation performance, we revisited the problem of separating overlapping nuclei. Previous studies have used algorithms such as intervening and concave contour-based normalized cut[41,42] on binary segmentation masks extracted using traditional segmentation methods such as Otsu’s method[8] to delineate overlapping nuclear boundaries. However, nuclear segmentation using traditional thresholding approaches failed to detect half of the nuclei in the Kaggle dataset (S2 Table), indicating that this approach is only effective for images with clean backgrounds and uniform signal. Recent studies have trained deep neural networks to learn the Euclidean distance transform (EDT) of the original mask corresponding to the input images[25,26], and apply a watershed transform on the model-predicted distance map to perform the final segmentation. This method has been further improved by adding the cell location information to the watershed transform to achieve better segmentation results.[26] These methods successfully address the challenges of separating overlapping objects, as EDT provides the neural networks with more morphological information.

**Fig 3:**
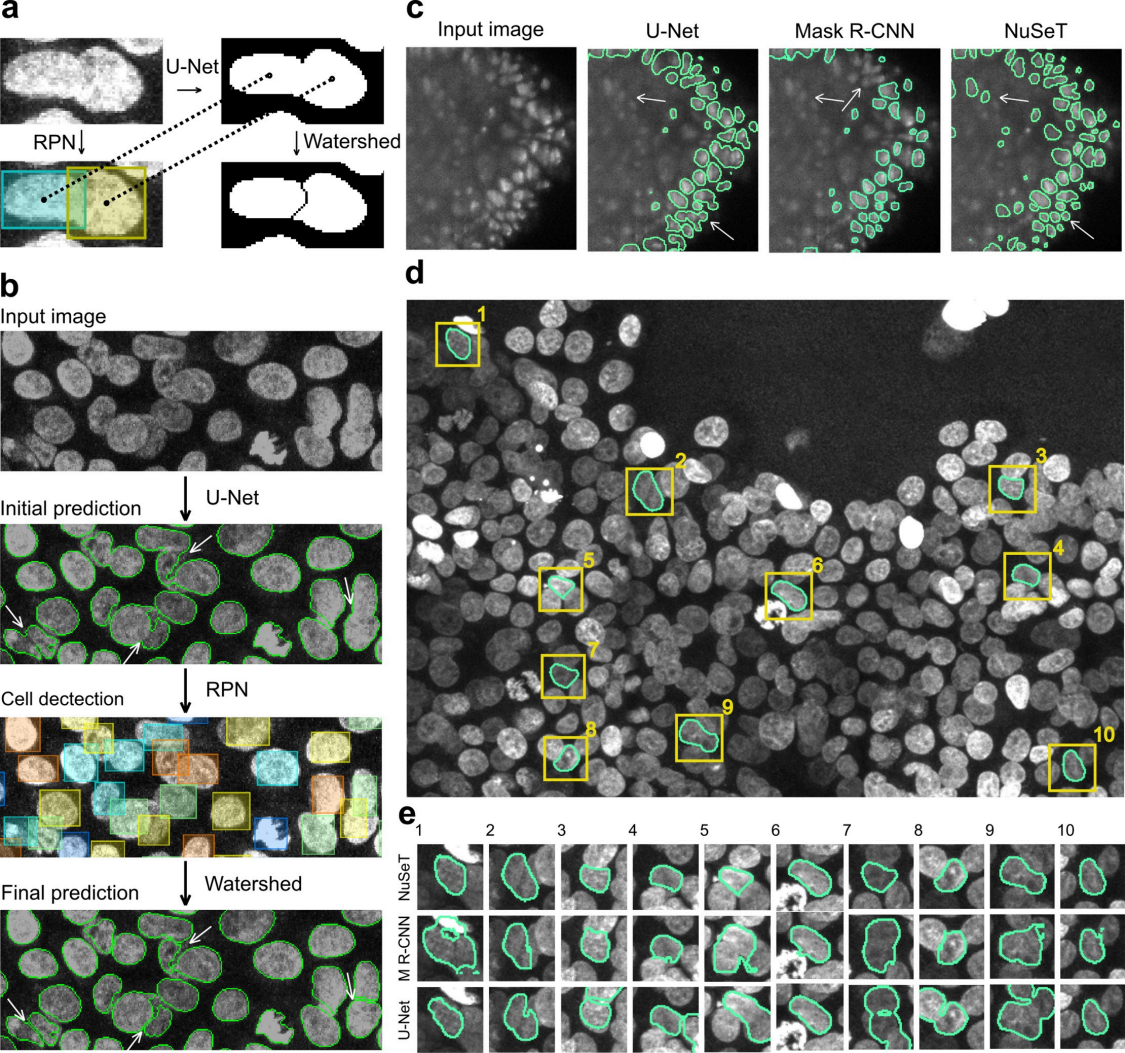
NuSeT efficiently addresses common segmentation challenges. **(A)** Implementing RPN-aided watershed algorithm improves touching cell separation. Bounding boxes and segmentation masks are computed by RPN and U-Net. Then the estimated centroid of each cell is computed from the coordinates of the bounding box. The watershed line is then estimated based on the binary mask and centroids. **(B)** Sample results showing that RPN successfully detects most of the cells, and watershed lines further separate touching cells. **(C)** Representative examples showing NuSeT detected more nuclei and better separated touching nuclei compared to Mask R-CNN and U-Net. **(D,E)** Examples nuclear masks generated using NuSeT for an image with high nuclei density (D). Comparison with the corresponding masks generated by Mask R-CNN and U-Net show subtle as well as prominent irregularities in boundary delineation that are circumvented by NuSeT (E).

Instead of training the model on EDT space, we trained the U-Net module directly with the binary masks. We also employed our modified RPN approach to detect nuclei. The nuclear centroids estimated from the RPN derived bounding box coordinates were passed as seeds for the watershed algorithm to generate cuts at touching nuclei boundaries on the U-Net produced binary masks (Fig 3A).[35,36]

Our results suggest that a modified RPN can detect most nuclei in overlapping regions, and a RPN-aided watershed separates 72%/94% of overlapping nuclei for Kaggle/ MCF10A dataset (Fig 3B, S2 Table). Compared with the modified RPN model without watershed, RPN-aided-watershed improved the overlapping nuclei separation performance and lowered the number of merge errors (S2 Table).

Through the integration of synthetic images, foreground normalization, and RPN-aided watershed, NuSeT consistently outperforms other state-of-the-art segmentation methods including U-Net and Mask R-CNN in nuclear boundary demarcation, particularly for blurry, low SNR nuclei (Fig 3C, S4 Fig, S2 Table). Mask R-CNN and NuSeT perform comparably in relatively sparse and homogenous samples (S2 Table). However, NuSeT approximates ground-truth boundaries more closely than U-Net and Mask R-CNN in samples with high cell densities (Fig 3D and 3E).

### Three-dimensional spatio-temporal tracking of individual nuclei in mammary acini

**Fig 4:**
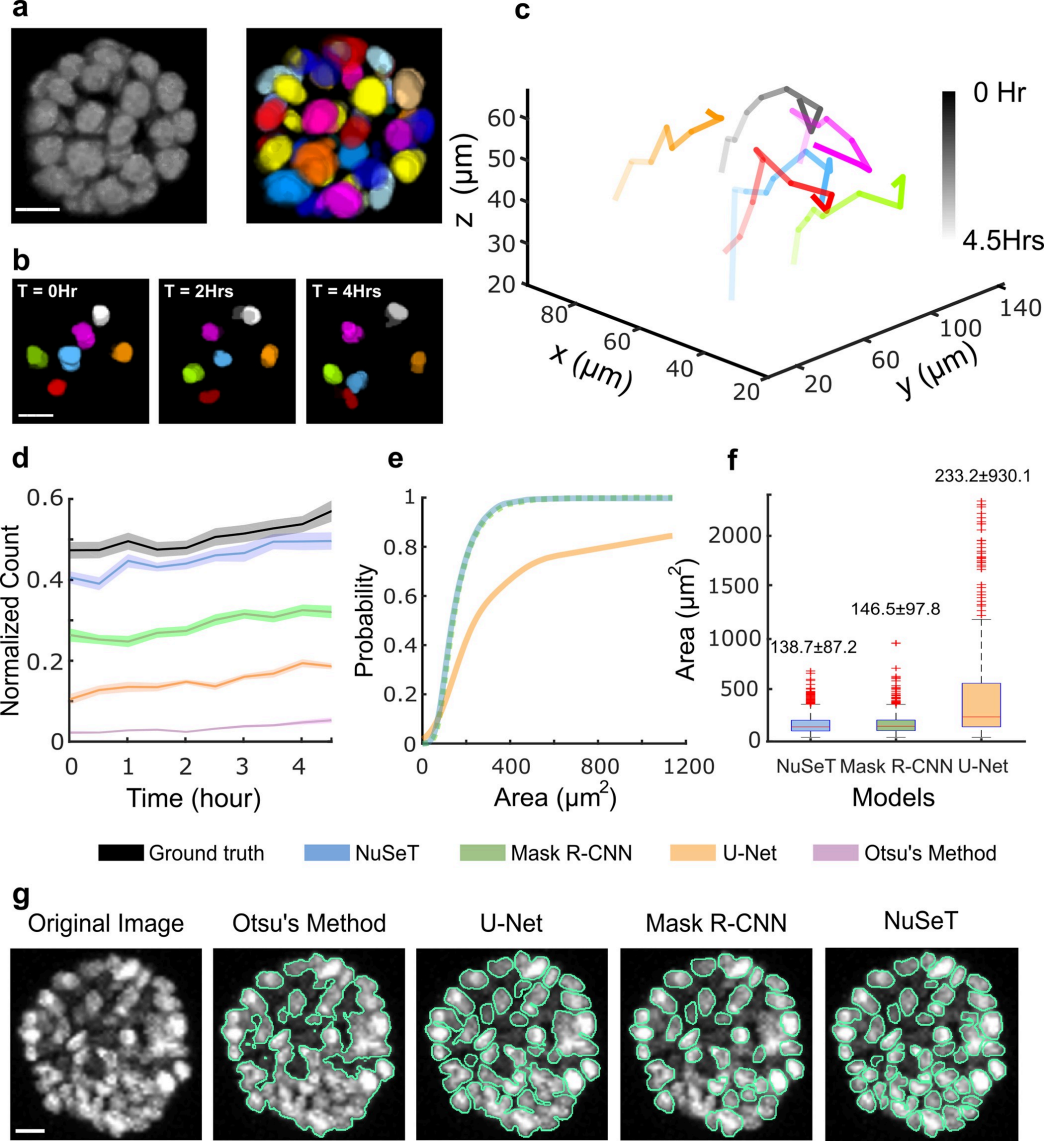
NuSeT effectively segments single nuclei in disorganizing dense mammary acini. (**A)** Representative 3D MCF10AT acinus segmentation using NuSeT. **(B)** Nuclei tracking. For ease of visualization, only a few of the segmented nuclei are shown at different time points. **(C)** 3D tracks of the nuclei shown in (**B**) over time, from 0 h (dark) to 4.5 h (light). **(D)** Number of nuclei detected in disorganizing acini at different time points using different segmentation methods. Data were collected from 8 representative acini and were normalized by the total number of nuclei at the last time point. Data from the first 5 hours are shown. **(E)** Cumulative distribution function plots of area of nuclei segmented using different methods. **(F)** Box plots of nuclear area distribution. The median area for each method is indicated on the top. The area box plot for Otsu’s method (median area: 2816.6 ± 2845 μm^2^) is shown in S6 Fig. **(G)** Representative examples comparing nuclei segmentation in dense mammary acini using different methods. Scale bars are 20 μm.

To investigate the performance of our algorithm in segmenting densely packed nuclei, we used NuSeT to segment and track nuclei in 3D reconstituted mammary acini grown from a Ras transformed MCF10A (MCF10AT) cell line. MCF10AT was chosen since upon continued growth in matrigel, this cell line produces mammary acini with very high cell density. Three-dimensional segmentation was performed by processing individual 2D slices from a Confocal Microscope Z-stack followed by three-dimensional reconstruction. NuSeT successfully segmented most of the nuclei in an acinus (Fig 4A), which facilitated seamless tracking of nuclei in mammary acini disorganizing on a 3D collagen matrix (Fig 4B and 4C). Both NuSeT and Mask R-CNN performed similarly on early-stage mammary acini (cell count = ~34 cells/acinus) (S5 Fig). To further evaluate the performance of different algorithms (NuSeT, U-Net, Mask R-CNN and Otsu’s method) on segmenting nuclei in mammary acini, we carried out nuclear segmentation on 2D projections of dense mammary acini.

NuSeT accurately segmented most of the nuclei in dense mammary acini (Fig 4D–4G). We were also able to track single nuclei through the entire process of acinar disorganization (*time* = 17.5 h, S5 Fig). Compared with the other widely used segmentation models, NuSeT performed consistently better at matching the number of nuclei detected manually for multiple acini (n = 8) at different stages of disorganization. U-Net, Otsu’s Method and Mask R-CNN all detected only a fraction of all the nuclei (Fig 4D) in a dense acinus. The distribution of areas of segmented nuclei (n = 1365 nuclei) across multiple acini (n = 8) at first 5 time points shows that while Mask R-CNN and NuSeT achieved comparable accuracy in nuclear boundary determination (median area of detected nuclei: 147 μm^2^ vs. 139 μm^2^, Fig 4E and 4F), Mask R-CNN only detected a subset of all nuclei (Fig 4D and 4G). Nuclear segmentation with U-Net on the other hand resulted in much larger nuclear area (median area of detected nuclei = 233 μm^2^, Fig 4E and 4F), indicating that U-Net often failed to separate touching nuclei (Fig 4G). All deep learning approaches outperformed the ‘traditional’ algorithm (Otsu’s Method, nuclei area = 2816.6 μm^2^, S5 Fig), as it rarely segmented single nuclei in dense settings (Fig 4G). Together, our results suggest that NuSeT outperforms both Mask R-CNN and U-Net in detecting nuclei and assigning boundaries for overlapping nuclei.

### Segmentation of histopathology samples and dividing cells

To further validate the performance and assess the generalizability of our algorithm, we extended NuSeT based segmentation to histopathology samples and rare-event detections as in the case of dividing cells.

**Fig 5:**
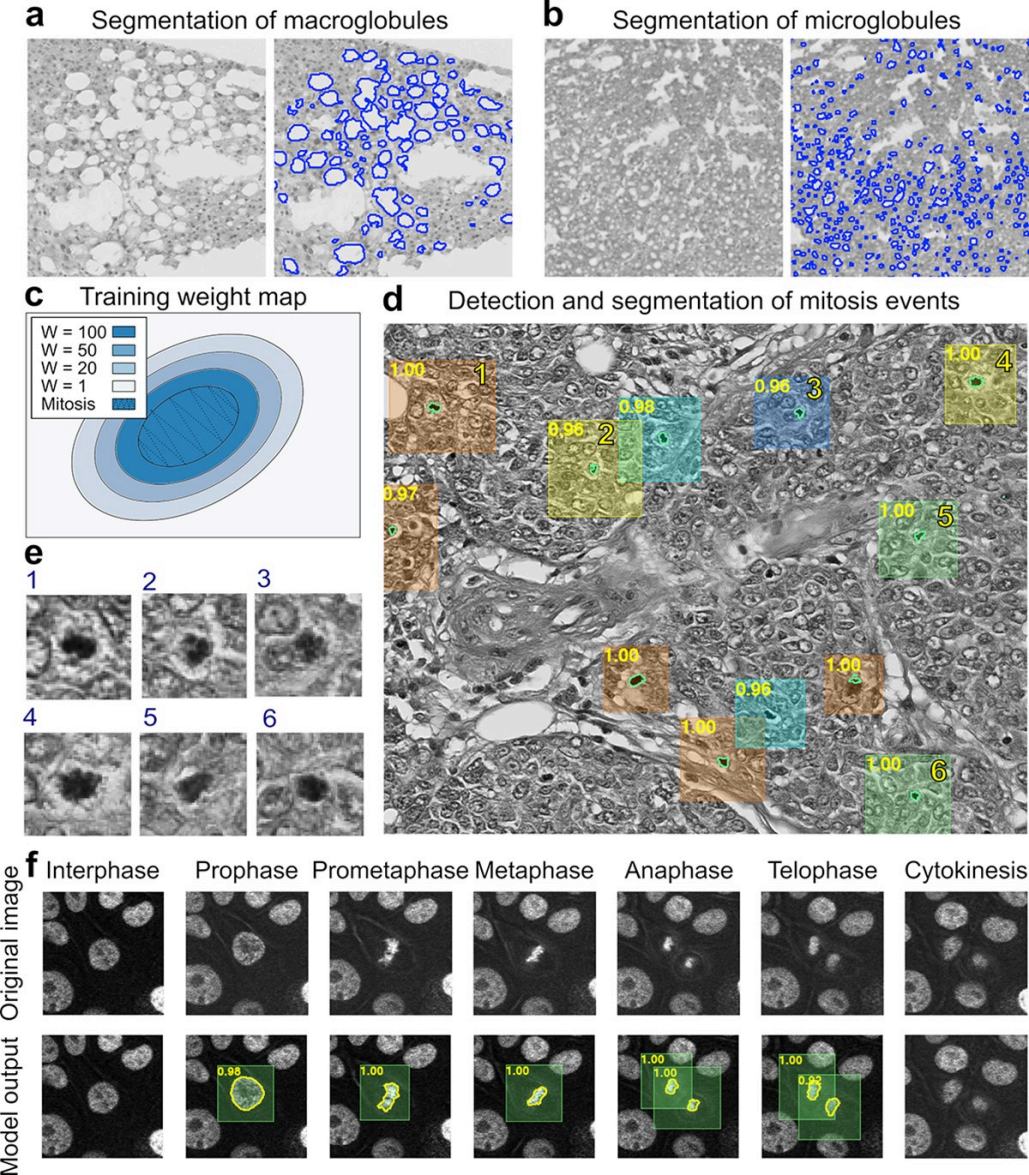
NuSeT can be applied to segment histopathology samples and detect mitotic events. **(A, B)** Representative example of liver fat globule segmentation using NuSeT. Notice that NuSeT performs well on both macroglobule **(A)** and microglobule **(B)** segmentation. **(C-E)**: Representative example of segmentation and detection of mitosis in breast cancer samples from ICPR 2012. **(C)** Weight map used for training the mitosis model. An ‘attention’ strategy has been used to focus more on the mitosis events and the environment surrounding them. The shaded region denotes the label for the mitotic event, and colors denote the weights applied during training process. **(D)** Representative example of mitosis detection and segmentation results with breast cancer sample. Scores on the top-left corner of the bounding boxes denote the possibility of a mitosis event evaluated from the model. Zoom-in of some detected mitotic events are shown in **(E)**. **(F)** Representative example of mitosis detection and segmentation results with fluorescent nuclei in a time lapse movie of MCF10A epithelial cells stably expressing histone H2B-eGFP. NuSeT can detect mitotic progression from prophase to telophase (mitotic events were identified by NuSeT and then manually classified into different phases).

As a test case for segmentation of histological samples, we re-trained NuSeT to segment fat globules in H & E stained sections of liver tissue. Evaluation of liver steatosis is a key step in both fatty liver disease diagnosis as well as pre and post-liver transplantation evaluation. The key challenges of segmenting fat globules from liver sections include detecting multi-scale globules and distinguishing them from tissue tearing artifacts. NuSeT successfully segmented both micro and macro-globules and avoided false detection of tissue tearing artifacts (Fig 5A and 5B), with mean IoU = 0.73 on a validation dataset.

Detecting and segmenting rare events in images are more challenging, as the majority area of the image is denoted as background, and back-propagation of gradients will overwhelm the model in classifying simple background pixels. Mitotic events, especially in images populated densely with non-dividing cells, is an example of such rare-event detection. To address this challenge, we designed an approach to highlight the regions close to mitotic events and give them more weights to ‘catch’ the attention of the model during training (Fig 5C). Using this strategy, we retrained the NuSeT model to detect and segment mitotic nuclei in human breast cancer histopathology samples[43,44], as the total number of mitotic events detected is a crucial indicator of the degree of malignancy for breast cancer diagnosis. Our results indicate that NuSeT can detect and segment the majority of mitosis events in breast cancer histopathology slides (Fig 5D and 5E), and was able to provide confidence scores for all the detected mitotic events (detection precision = 56.22%, recall = 58.85% on validation dataset). When we inspected the data, we found several detection errors stemming from mis-classification of other objects such as dense nuclei and lymphocytes, which are very similar in appearance to mitotic nuclei. When trained with fluorescently labeled nuclei (MCF10A cells stably expressing the nuclear marker histone H2B-eGFP), NuSeT captured the mitotic progression from prophase to telophase (Fig 5D and 5E, S7 Fig) (detection precision = 73.90%, recall = 90.20% on validation dataset). Together our results indicate that NuSeT is highly generalizable and can be applied to histopathology segmentation tasks as well as detection of rare events in samples of high clinical value.

## Discussion

Here we present a deep learning model for nuclear segmentation that is robust to a wide range of image variabilities. Compared with previous models that need to be trained separately for specific cell types, NuSeT provides a more generalized approach for segmenting fluorescent nuclei varying in size, brightness and density. We have also developed novel training and pre/post-processing approaches to address common problems in biological image processing. Our results indicate that every stage in deep learning, from data collection to post-processing, is crucial to training an accurate and robust nuclear segmentation model. When compared with the state-of-the-art cell segmentation models, NuSeT separates touching nuclei better than U-Net and detects more nuclei than Mask R-CNN. Thus, it assimilates the advantages of both semantic segmentation (U-Net) and instance segmentation (Mask R-CNN) and circumvents their limitations. This combination enables NuSeT to analyze complex three-dimensional cell clusters such as mammary acini and track single nuclei in dynamic crowded environments. When retrained on histopathology images, NuSeT is able to segment cells and rare events in H&E Stained samples using new training data. Therefore, we expect NuSeT to find wide applicability, particularly in the areas of cell lineage tracing and clinical diagnosis.

Although we have modified the original RPN architecture to adjust detection scales based on the median nucleus size for each image, NuSeT assumes similar nuclear sizes in the same image. This may account for the occasional errors in nuclei segmentation when using RPN-aided watershed. If markedly irregular (such as dim/deformed/blurry) nuclei are encountered in the same image, RPN may over- or under-detect the nuclei and produce incorrect numbers of bounding boxes. This would lead to marker misplacement and erroneous segmentation lines. While we expect NuSeT to perform well for nuclei of most mammalian cell types, its performance for mixed populations remains to be validated. Recent studies have extracted image features from multi-scale and ‘pyramidal hierarchy’ neural networks to improve detection accuracy for objects with large size variations.[45,46] Subsequent work has improved object detection in dense samples using weighted loss functions.[27] By incorporating these advances into our current model, we expect to further improve NuSeT in multi-scale nuclei detection.

Our approach has cross-platform support and comparatively low hardware requirements (S5 Table). With a medium-level Nvidia GPU (Quadro P4000), training an accurate model only takes five hours, and the inference proceeds at 1.98 seconds/Mega pixel. From a user standpoint, the NuSeT GUI enables researchers to easily segment their images without needing to understand all the details of machine-learning, which connects state-of-the-art computer vision algorithms to a suite of cell biology problems. While in the present work we provide an effective and efficient pipeline for cell nuclei segmentation, this approach should be easily adaptable to a wide variety of image segmentation tasks involving densely packed and overlapping objects, such as jumbled piles of boxes or people in crowds.

## Methods

### Kaggle dataset preprocessing

The Kaggle dataset was downloaded from the Broad Bioimage Benchmark Collection (Accession number BBBC038v1).[33] This dataset was sampled from a wide range of organisms include human, mice and flies, and the nuclei were recorded under different imaging conditions. Stage-1 training and test datasets were used for training and validation process. All the images were manually censored and training data with low segmentation accuracies were discarded. Only fluorescent images were used for training and validation process. We converted the run-length encoded labels to binary masks for both training and validation labels in MATLAB. The final Kaggle dataset used for our model contains 543 images for training and 53 images for validation. Segmentation errors, including mask misalignment and touching cells, were manually corrected image-by-image for training and validating data.

### Mammary acini, MCF10A monolayer growth, and mitosis data collection

The MCF10A data and fluorescent mitosis data were collected on an Olympus FV10i confocal microscope with a 60X objective on MCF10A human breast epithelial cell line. The cell nuclei were stained with 1uM Sir-DNA for 1 hour before imaging. The test set consists of 25 experiments with the corresponding ground-truth binary labels. MCF10AT acini were grown and the acini disorganization assays were performed as described in Shi *et al*.[4] The fluorescent mitosis data were collected on an Olympus FV10i confocal microscope with a 60X objective on MCF10A human breast epithelial cell line stably expressing H2B-eGFP with 10 minutes time interval over 3.5 days.[4] The final fluorescent mitosis training dataset used for our model contains 518 images for training and 57 images for validation.

### Liver tissue slide collection

Biopsied liver tissue slides were stained with hematoxylin and eosin and scanned with Philips Intellisite Pathology Solutions or Aperio AT2 scanners. To accelerate the training process, each liver slide was down-sized by 8 folds and partitioned into 20–30 tiles of dimensions 256 pixels by 256 pixels. The fat globules were manually annotated by a pathologist for both training and validation datasets. The final training dataset used for our model contains 247 images for training and 10 images for validation.

### Histopathology mitosis dataset preprocessing

The Mitosis dataset was downloaded from ICPR 2012[43] and ICPR 2014[44] mitosis detection contests. Breast cancer biopsy slides ranging from low-grade atypia to high-grade atypia were stained with hematoxylin and eosin and scanned by two scanners: Aperio Scanscope XT and Hamamatsu Nanozoomer 2.0-HT. Mitosis events were annotated by at least two individual pathologists. Training datasets acquired at 40X magnifications from ICPR 2012 and 2014 were used for training the model. All the images were manually censored and training data without any mitosis events were excluded. We also converted the coordinates of mitosis locations into binary masks for both training and validation labels using MATLAB scripts. The final training dataset used for our model contains 621 images for training and 69 images for validation. To accelerate the training, we down-sampled the original images by a factor of 2. The trained model was further tested with ICPR 2012 test dataset.

### Data augmentation

To accelerate the training process, only simple data augmentation techniques were applied to the training images. We adopted mirror flip and small rotation (10 degrees, counterclockwise) for training data to alleviate the overfitting problem.

### Synthetic data generation

Synthetic cell nuclei images were generated by utilizing nuclei-like blobs (adapted from https://stackoverflow.com/questions/3587704/good-way-to-procedurally-generate-a-blob-graphic-in-2d), as well as random shape polygons/lines. Signal (brightness) variations were added to both blobs and polygons/lines. The sizes of nuclei like blobs, polygons and lines were varied image-by-image to simulate different imaging conditions. The synthetic images were generated with various image sizes, with width and height ranging from 256 pixels to 640 pixels. Gaussian noise and Gaussian blur were added to these images. We applied overlapping of blobs to strengthen the model capability in separating touching nuclei. The binary masks of the synthetic images were generated separately. To correctly separate all overlapping blobs in the corresponding segmentation masks, the positions of blobs were used as markers to apply watershed transform[36] on overlapping blobs.

### Training and inference details

To construct the training data, we incorporated 543 training images from the Kaggle dataset and 25 training images from MCF10A dataset as the base-training dataset. After data augmentation, the training set contained 568 (original) + 568 (flip) + 568 (rotate) = 1704 images. Then we mixed the real images with synthetic images at 1:1 ratio to generate the final training dataset. The training images were normalized by subtracting the foreground mean value and dividing by the foreground standard deviation. Since U-Net contains 4 down-sampling and up-sampling layers, to make the tensors at each layer compatible, training images were further cropped so that widths and heights of the images were adjusted to the nearest multiple of 16. To train RPN, the ground truth coordinates for bounding boxes were calculated based on the binary nuclei masks. The coordinates of the bounding box, (x_min, y_min), (x_max, y_max) were denoted as the most upper-left and lower-right pixels of the corresponding nuclei. Weight matrices were calculated per mask with w0 = 10 and sigma^2^ = 5 pixels. To avoid out-of-memory, one image was fed into the network at a time. During the training, the sequence order of the training data was reshuffled before each epoch to prevent overfitting. The learning rate was set to 5e-5, and Rmsprop[47] was utilized as the training optimizer, and the best performance model was chosen within the first 30 epochs. The training loss was the sum of segmentation loss and detection loss. Segmentation loss was the sum of binary cross-entropy loss[10] and Dice loss, and the detection loss was the class loss and regression loss as described in previous work.[18]

Two validation datasets were used to benchmark the model performance. The Kaggle validation dataset[33] contains 50 images that have various types of nuclei under different imaging conditions. The MCF10A dataset contains 25 images that have homogenous nuclei imaged under the same setting manner. This study was performed on Nvidia Quadro P4000. Additional segmentation performance is shown in S6 Fig.

### Model evaluation

Eight models were chosen to compare their performance on both Kaggle and MCF10A validation dataset, including Otsu’s Method[8], Deep Cell 1.0[14], U-Net[15], Mask R-CNN[19], NuSeT with whole-image normalization and without synthetic data, NuSeT with whole-image normalization, NuSeT with foreground normalization, and NuSeT with foreground normalization and RPN-aided watershed. The entire training dataset (with data augmentation and synthetic images) was applied to train all NuSeT models. To test Deep Cell 1.0’s performance on the Kaggle and MCF10A dataset, we selected the HeLa fluorescent nuclei model from the initial set of models from (http://www.deepcell.org/predict, accessed on Feb 25^th^, 2019). Since no pre-trained two-dimensional fluorescent nuclei segmentation model was found from U-Net[15,34], we trained U-Net on our training dataset (without synthetic data) as our closest estimate for performance. The original Mask R-CNN model was trained for real-life segmentations. Therefore we trained Mask R-CNN on our training dataset (without synthetic data) starting from FPN-101 backbone.[48] We did not apply the aforementioned modified RPN to Mask R-CNN, since Mask R-CNN performs the segmentation strictly after the RPN detection, effectively blocking information transfer between the detection and the segmentation modules. We removed cells smaller than 1/5 of the average cell area in the image for prediction masks from all models prior to benchmarking.

To evaluate model performance, we adopted the following performance metrics: percentage of touching cell separated, correct detections, incorrect detections, split errors, merge errors, catastrophe errors, false negative detection rate (F.N. rate), false positive detection rate (F.P. rate), mean I.U., RMSE, F1 and pixel accuracy. The first eight metrics were evaluated on the nuclei level, and the last four metrics indicate the performance on the pixel-level. The calculation of correct and incorrect detections, as well as split, merge and catastrophe errors have been described in previous works.[29,30] Briefly, correct detections denote the number of predicted cells that can link with ground truth cells, and incorrect detections refer to the number of unlinked cells from the prediction. Split, merge and catastrophe errors are subsets of incorrect detections, where split and merge errors describe the splitting and merging of ground truth cells into prediction cells, and catastrophe errors refer to the uneven matching of ground truth and prediction cells.[29, 30] The percentage of touching nuclei separated is calculated as:

$${\%}_{nuclei\;separated}=\frac{N_{nuclei\;separated}}{N_{total\;overlapping\;nuclei}}$$

*N*_*nuclei separated*_ denotes the number of touching nuclei that have been successfully separated by the model, *N*_*total overlapping nuclei*_ denotes the total number of touching nuclei in the entire dataset.

F.N. rate is the proportion of the nuclei that the model fails to detect in the entire dataset. The detection failure is defined as: given a nucleus’ ground-truth binary mask, find the corresponding model-predicted mask that has the largest overlap ratio, which is measured by:

$$overlap\;ratio=\frac{A_{GT}\cap A_{pred}}{A_{GT}}$$

Where *A*_*GT*_ is the area of ground truth nucleus, *A*_*pred*_ is the area of model-predicted nucleus. If the overlap ratio is smaller than 0.7, it is suggested that the model fails to detect the nucleus. Hence the F.N. rate is denoted as:

$$F.N.rate=\frac{N_{missing\;nuclei}}{N_{total\;nuclei}}$$

*N*_*missing nuclei*_ denotes the number of nuclei that the model fails to detect. *N*_*total nuclei*_ denotes the total number of nuclei labelled by ground-truth in the dataset.

Likewise, F.P. rate is the proportion of the nuclei that the model mis-detects in the entire dataset. The mis-detection is defined as: given a nucleus’ model-predicted mask, find the corresponding ground-truth mask that has the largest overlap, if the overlap ratio of the model predicted mask and the ground-truth mask is smaller than 0.7, it is suggested that the model detects an ‘nucleus’ that does not exist in the ground-truth. Hence the F.P. rate is denoted as:

$$F.P.rate=\frac{N_{mis-detections}}{N_{total\;nuclei}}$$

*N*_*mis−detections*_ denotes the number of model-predicted nuclei that found no match in the ground-truth labels.

Pixel-level metrics mean IU, F1, RMSE and pixel accuracy were calculated as:

$$mean\;IU=\frac{1}{N_{cls}}\sum_{n}\frac{{TP}_{n}}{{TP}_{n}+{FN}_{n}+{FP}_{n}}$$

$$F1=\frac{1}{N_{cls}}\sum_{n}\frac{{2TP}_{n}}{{2TP}_{n}+{FN}_{n}+{FP}_{n}}$$

$$RMSE=\sqrt[{2}]{\frac{1}{N_{pix}}\sum_{i\in I}{\left(y_{i_{pred}}-y_{i}\right)}^{2}}$$

$$pixel\;accuracy=\frac{TP+TN}{TP+TN+FP+FN}$$

Where *TP*, *TN*, *FP*, *FN* denotes the pixel-level counts of true positive, true negative, false positive and false negative for single image. *N*_*cls*_ denotes the number of classes a pixel can be predicted to, in our case *N*_*cls*_ = 2 (foreground and background), and *TP*_*n*_ denotes the true positive counts of class n. *N*_*pix*_ is the number of pixels in the image, and $y_{i_{pred}}$ is the binary value of pixel i in the model-predicted mask, *y*_*i*_ is the binary value of pixel i in the ground-truth mask. The pixel-level metrics over the entire dataset were then calculated as the average metrics of all the images in the dataset. Precision and recall were calculated as *TP*/(*TP*+*FP*) and *TP*/(*TP*+*FN*).

## Supporting information

S1 Table Internal performance comparison across different datasets. Step-by-step addition of synthetic data, foreground normalization, and RPN-aided watershed result in better performance at object-level. Notice that the pixel-level accuracies (mean IU, RMSE, F1, pixel accuracy) are similar, despite marked differences in object-level metrics.
(DOCX)

S2 Table External performance comparison of published models across different datasets. (DOCX)

S3 Table Effects of whole-image normalization model accuracy on the performance of the foreground normalization model. Whole-image normalization models were trained to different mean IoU levels and connected to the same foreground model to benchmark the final model performance. Metrics were evaluated from three individual experiments.
(DOCX)

S4 Table Effects of adding foreground normalization on different models. Comparison of segmentation performance for NuSeT, UNet, Mask R-CNN trained with per-image normalization and foreground normalization. Foreground normalization consistently improves the segmentation performance on most object-level metrics for both NuSeT and UNet.
(DOCX)

S5 Table Memory footprint, training and inference speed comparison for different models. (DOCX)

S1 Fig Common problems encountered in nuclei segmentation. Some common factors that affect the quality of nuclei segmentation, are, touching cells (**A**), signal variation (**B**), sample preparation artifacts and contaminants (**C**), and low signal to noise ratio (**D**). Colored outlines represent the goals (ground truth) for segmentation tasks.
(TIF)

S2 Fig Adjusting bounding box dimensions based on nuclear size. Historically RPN has used a set of rigid base sizes for all bounding boxes, which resulted in high detection error rate in the Kaggle dataset. We improved the RPN so that it applies different bounding box base sizes for different images. The base size is determined by the median of all nuclei sizes within the image. Nuclei sizes are defined by the maximum value between nuclei widths and heights.
(TIF)

S3 Fig Foreground normalization is more robust than whole-image normalization in handling images with sample preparation artifacts. Normalizing samples with or without sample artifacts using different normalization methods show that images have more consistent nuclei signals after foreground normalization (highlighted by arrows).
(TIF)

S4 Fig Additional segmentation performance comparisons across algorithms, including traditional thresholding approach (Otsu’s method) and Deep Cell 1.0. (TIF)

S5 Fig Additional mammary acini segmentation and tracking results. (**A)** Three-dimensional acini tracking with different deep-learning models. **(B)** Additional time-lapse tracking of selected nuclei. **(C)** Comparison of nuclei area distribution for Otsu’s method (median area: 2816.6 ± 2845.0 μm^2^) and NuSeT (median area: 138.7 ± 87.2 μm^2^).
(TIF)

S6 Fig Additional examples showing NuSeT’s performance when handling images with signal variations, shape variations, touching nuclei and sample preparation artifacts. (TIF)

S7 Fig Additional fluorescent mitotic events detection and segmentation results. (TIF)

S1 Text Supplementary notes about the NuSeT user interface (UI). (DOCX)
