# Supplementary material for: NuSeT: A deep learning tool for reliably separating and analyzing crowded cells
Source: PLoS Comput Biol. 2020 Sep 14;16(9):e1008193. doi: 10.1371/journal.pcbi.1008193 (PMC7515182; doi:10.1371/journal.pcbi.1008193)
Supplement: S1 Table — Step-by-step addition of synthetic data, foreground normalization, and RPN-aided watershed result in better performance at object-level. Notice that the pixel-level accuracies (mean IU, RMSE, F1, pixel accuracy) are similar, despite marked differences in object-level metrics. (DOCX) [file pcbi.1008193.s001.docx]

**S1 Table. Internal performance comparison across different datasets.**

Kaggle fluorescent dataset (object-level metrics)

| Whole-image Norm. | Synthetic data | Foreground Norm. | Watershed | % of overlapping cells separated | Correct  detections | Incorrect  detections | Splits | Merges | Catastrophes | FN rate | FP rate |
| --- | --- | --- | --- | --- | --- | --- | --- | --- | --- | --- | --- |
| √ |  |  |  | 37.70% | 1715 | 420 | 16 | 220 | 6 | 16.74% | 3.38% |
| √ | √ |  |  | 51.78% | 1810 | 440 | 30 | 167 | 10 | 18.57% | 4.62% |
|  | √ | √ |  | 65.09% | 1946 | 441 | 41 | 130 | 17 | 15.83% | 4.43% |
|  | √ | √ | √ | 72.13% | 1960 | 443 | 42 | 118 | 18 | 16.47% | 4.47% |

Kaggle fluorescent dataset (pixel-level metrics)

| Whole-image Norm. | Synthetic data | Foreground Norm. | Watershed | Mean IoU | RMSE | F1 | Pixel accuracy |
| --- | --- | --- | --- | --- | --- | --- | --- |
| √ |  |  |  | 0.88 | 0.16 | 0.93 | 0.96 |
| √ | √ |  |  | 0.88 | 0.16 | 0.93 | 0.96 |
|  | √ | √ |  | 0.89 | 0.15 | 0.94 | 0.97 |
|  | √ | √ | √ | 0.88 | 0.16 | 0.93 | 0.97 |

MCF10A dataset (object-level metrics)

| Whole-image Norm. | Synthetic data | Foreground Norm. | Watershed | % of overlapping cells separated | Correct  detections | Incorrect  detections | Splits | Merges | Catastrophes | FN rate | FP rate |
| --- | --- | --- | --- | --- | --- | --- | --- | --- | --- | --- | --- |
| √ |  |  |  | 72.42% | 3056 | 223 | 5 | 105 | 2 | 5.25% | 3.08% |
| √ | √ |  |  | 81.58% | 3125 | 185 | 3 | 68 | 0 | 5.97% | 2.68% |
|  | √ | √ |  | 92.03% | 3156 | 187 | 7 | 39 | 1 | 7.79% | 2.54% |
|  | √ | √ | √ | 93.67% | 3137 | 180 | 7 | 33 | 1 | 8.68% | 2.51% |

MCF10A dataset (pixel-level metrics)

| Whole-image Norm. | Synthetic data | Foreground Norm. | Watershed | Mean IoU | RMSE | F1 | Pixel accuracy |
| --- | --- | --- | --- | --- | --- | --- | --- |
| √ |  |  |  | 0.93 | 0.19 | 0.96 | 0.96 |
| √ | √ |  |  | 0.94 | 0.17 | 0.97 | 0.97 |
|  | √ | √ |  | 0.93 | 0.18 | 0.96 | 0.96 |
|  | √ | √ | √ | 0.92 | 0.19 | 0.96 | 0.96 |

Step-by-step addition of synthetic data, foreground normalization, and RPN-aided watershed result in better performance at object-level. Notice that the pixel-level accuracies (mean IU, RMSE, F1, pixel accuracy) are similar, despite marked differences in object-level metrics.
