# Supplementary material for: NuSeT: A deep learning tool for reliably separating and analyzing crowded cells
Source: PLoS Comput Biol. 2020 Sep 14;16(9):e1008193. doi: 10.1371/journal.pcbi.1008193 (PMC7515182; doi:10.1371/journal.pcbi.1008193)
Supplement: S2 Table — (DOCX) [file pcbi.1008193.s002.docx]

**S2 Table. External performance comparison of published models across different datasets**.

Kaggle fluorescent dataset (object-level metrics)

|  | % of overlapping cells separated | Correct  detections | Incorrect  detections | Splits | Merges | Catastrophes | FN rate | FP rate |
| --- | --- | --- | --- | --- | --- | --- | --- | --- |
| Otsu’s Method | 11.37% | 818 | 1507 | 7 | 267 | 6 | 51.26% | 0.53% |
| Deep Cell 1.0 | 24.11% | 1372 | 970 | 70 | 265 | 38 | 4.88% | 29.27% |
| U-Net | 40.02% | 1794 | 356 | 13 | 197 | 7 | 13.13% | 3.79% |
| Mask R-CNN | 80.04% | 1919 | 402 | 35 | 68 | 17 | 25.33% | 4.05% |
| NuSeT | 65.09% | 1946 | 441 | 41 | 130 | 17 | 15.83% | 4.43% |
| NuSeT+ watershed | 72.13% | 1960 | 443 | 42 | 118 | 18 | 16.47% | 4.47% |

Kaggle fluorescent dataset (pixel-level metrics)

|  | mean IU | RMSE | F1 | Pixel accuracy |
| --- | --- | --- | --- | --- |
| Otsu’s Method | 0.78 | 0.22 | 0.86 | 0.93 |
| Deep Cell 1.0 | 0.79 | 0.21 | 0.87 | 0.94 |
| U-Net | 0.89 | 0.15 | 0.94 | 0.97 |
| Mask R-CNN | 0.85 | 0.18 | 0.92 | 0.96 |
| NuSeT | 0.89 | 0.15 | 0.94 | 0.97 |
| NuSeT+ watershed | 0.88 | 0.16­ | 0.93 | 0.97 |

MCF10A dataset (object-level metrics)

|  | % of overlapping cells separated | Correct  detections | Incorrect  detections | Splits | Merges | Catastrophes | FN rate | FP rate |
| --- | --- | --- | --- | --- | --- | --- | --- | --- |
| Otsu’s Method | 31.55% | 2036 | 10476 | 47 | 314 | 8 | 20.13% | 6.54% |
| Deep Cell 1.0 | 89.15% | 2968 | 302 | 22 | 49 | 3 | 10.59% | 4.11% |
| U-Net | 84.96% | 3150 | 160 | 3 | 63 | 1 | 5.57% | 2.28% |
| Mask R-CNN | 95.65% | 3233 | 198 | 9 | 15 | 2 | 4.77% | 4.62% |
| NuSeT | 92.03% | 3156 | 187 | 7 | 39 | 1 | 7.79% | 2.54% |
| NuSeT+ watershed | 93.67% | 3137 | 180 | 7 | 33 | 1 | 8.68% | 2.51% |

MCF10A dataset (pixel-level metrics)

|  | mean IU | RMSE | F1 | Pixel accuracy |
| --- | --- | --- | --- | --- |
| Otsu’s Method | 0.82 | 0.30 | 0.90 | 0.90 |
| Deep Cell 1.0 | 0.85 | 0.28 | 0.92 | 0.92 |
| U-Net | 0.94 | 0.17 | 0.97 | 0.97 |
| Mask R-CNN | 0.91 | 0.21 | 0.95 | 0.95 |
| NuSeT | 0.93 | 0.18 | 0.96 | 0.96 |
| NuSeT+ watershed | 0.92 | 0.19 | 0.96 | 0.96 |
