# Supplementary material for: NuSeT: A deep learning tool for reliably separating and analyzing crowded cells
Source: PLoS Comput Biol. 2020 Sep 14;16(9):e1008193. doi: 10.1371/journal.pcbi.1008193 (PMC7515182; doi:10.1371/journal.pcbi.1008193)
Supplement: S3 Table — Whole-image normalization models were trained to different mean IoU levels and connected to the same foreground model to benchmark the final model performance. Metrics were evaluated from three individual experiments. (DOCX) [file pcbi.1008193.s003.docx]

**S3 Table. Effects of whole-image normalization model accuracy on the performance of the foreground normalization model**

Kaggle fluorescent dataset (object-level metrics)

| Whole-image mean IoU range | % of overlapping cells separated | Correct  detections | Incorrect  detections | Splits | Merges | Catastrophes | FN rate | FP rate |
| --- | --- | --- | --- | --- | --- | --- | --- | --- |
| 0.66-0.68 | 80.87±6.73% | 1665±119 | 696±21 | 58±14 | 92±21 | 12±2 | 37.01±11.82% | 5.29±4.89% |
| 0.68-0.70 | 76.52±3.57% | 1815±119 | 528±59 | 52±12 | 104±15 | 14±3 | 27.18±10.54% | 4.53±2.04% |
| 0.70-0.72 | 80.30±3.40% | 1769±119 | 461±86 | 73±21 | 78±24 | 15±2 | 33.06±8.33% | 2.70±0.48% |
| 0.72-0.74 | 75.25±2.37% | 1866±51 | 518±27 | 60±24 | 98±28 | 18±1 | 23.09±6.12% | 4.62±1.27% |
| 0.74-0.76 | 75.09±1.68% | 1887±32 | 485±22 | 48±4 | 112±6 | 17±1 | 23.44±4.10% | 3.83±0.88% |
| 0.76-0.78 | 74.57±0.57% | 1897±5 | 482±1 | 44±2 | 115±2 | 16±1 | 22.94±1.22% | 3.62±0.15% |
| 0.78-0.80 | 73.32±1.59% | 1903±8 | 476±10 | 41±1 | 119±7 | 16±0 | 20.59±3.22% | 4.05±0.46% |
| 0.80-0.82 | 73.12±1.07% | 1936±19 | 473±13 | 42±1 | 118±5 | 17±2 | 18.52±0.95% | 4.04±0.34% |
| 0.82-0.84 | 71.91±0.20% | 1946±13 | 455±5 | 43±1 | 119±0 | 18±1 | 17.20±0.67% | 4.37±0.07% |
| 0.84-0.86 | 71.17±0.31% | 1934±10 | 454±4 | 43±0 | 122±3 | 18±2 | 16.83±0.41% | 4.42±0.22% |
| 0.86-0.88 | 70.97±0.34% | 1935±6 | 452±4 | 42±2 | 124±1 | 16±0 | 16.60±0.28% | 4.56±0.12% |
| 0.88-0.90 | 70.84±0.25% | 1930±1 | 457±3 | 42±0 | 124±3 | 17±2 | 16.60±0.42% | 4.79±0.34% |

Kaggle fluorescent dataset (pixel-level metrics)

| Whole-image mean IoU range | Mean IoU | RMSE | F1 | Pixel accuracy |
| --- | --- | --- | --- | --- |
| 0.66-0.68 | 0.82±0.03 | 0.19±0.00 | 0.88±0.03 | 0.95±0.00 |
| 0.68-0.70 | 0.84±0.01 | 0.18±0.00 | 0.90±0.01 | 0.96±0.00 |
| 0.70-0.72 | 0.85±0.01 | 0.17±0.01 | 0.91±0.00 | 0.95±0.00 |
| 0.72-0.74 | 0.85±0.01 | 0.17±0.00 | 0.91±0.01 | 0.96±0.00 |
| 0.74-0.76 | 0.86±0.01 | 0.17±0.00 | 0.92±0.01 | 0.96±0.00 |
| 0.76-0.78 | 0.86±0.01 | 0.17±0.00 | 0.92±0.01 | 0.96±0.00 |
| 0.78-0.80 | 0.86±0.01 | 0.17±0.00 | 0.91±0.01 | 0.96±0.00 |
| 0.80-0.82 | 0.87±0.01 | 0.16±0.00 | 0.93±0.00 | 0.96±0.00 |
| 0.82-0.84 | 0.88±0.00 | 0.16±0.00 | 0.93±0.00 | 0.96±0.00 |
| 0.84-0.86 | 0.88±0.00 | 0.16±0.00 | 0.93±0.00 | 0.96±0.00 |
| 0.86-0.88 | 0.88±0.00 | 0.16±0.00 | 0.93±0.00 | 0.96±0.00 |
| 0.88-0.90 | 0.88±0.00 | 0.16±0.00 | 0.93±0.00 | 0.96±0.00 |

Whole-image normalization models were trained to different mean IoU levels and connected to the same foreground model to benchmark the final model performance. Metrics were evaluated from three individual experiments.
