# Supplementary material for: NuSeT: A deep learning tool for reliably separating and analyzing crowded cells
Source: PLoS Comput Biol. 2020 Sep 14;16(9):e1008193. doi: 10.1371/journal.pcbi.1008193 (PMC7515182; doi:10.1371/journal.pcbi.1008193)
Supplement: S4 Table — Comparison of segmentation performance for NuSeT, UNet, Mask R-CNN trained with per-image normalization and foreground normalization. Foreground normalization consistently improves the segmentation performance on most object-level metrics for both NuSeT and UNet. (DOCX) [file pcbi.1008193.s004.docx]

**S4 Table. Effects of adding foreground normalization on different models.**

**NuSeT:**

Kaggle fluorescent dataset

|  | Object-level metrics | | | | | | | | Pixel-level metrics | | | |
| --- | --- | --- | --- | --- | --- | --- | --- | --- | --- | --- | --- | --- |
| Normalization  method | % of overlapping cells separated | Correct  detections | Incorrect  detections | Splits | Merges | Catastrophes | FN rate | FP rate | Mean IoU | RMSE | F1 | Pixel accuracy |
| Whole-image Norm. | 51.78% | 1810 | 440 | 30 | 167 | 10 | 18.57% | 4.62% | 0.88 | 0.16 | 0.93 | 0.96 |
| Foreground Norm. | 65.09% | 1946 | 441 | 41 | 130 | 17 | 15.83% | 4.43% | 0.89 | 0.15 | 0.94 | 0.97 |

MCF10A dataset

|  | Object-level metrics | | | | | | | | Pixel-level metrics | | | |
| --- | --- | --- | --- | --- | --- | --- | --- | --- | --- | --- | --- | --- |
| Normalization  method | % of overlapping cells separated | Correct  detections | Incorrect  detections | Splits | Merges | Catastrophes | FN rate | FP rate | Mean IoU | RMSE | F1 | Pixel accuracy |
| Whole-image Norm. | 81.58% | 3125 | 185 | 3 | 68 | 0 | 5.97% | 2.68% | 0.94 | 0.17 | 0.97 | 0.97 |
| Foreground Norm. | 92.03% | 3156 | 187 | 7 | 39 | 1 | 7.79% | 2.54% | 0.93 | 0.18 | 0.96 | 0.96 |

**UNet:**

Kaggle fluorescent dataset

|  | Object-level metrics | | | | | | | | Pixel-level metrics | | | |
| --- | --- | --- | --- | --- | --- | --- | --- | --- | --- | --- | --- | --- |
| Normalization  method | % of overlapping cells separated | Correct  detections | Incorrect  detections | Splits | Merges | Catastrophes | FN rate | FP rate | Mean IoU | RMSE | F1 | Pixel accuracy |
| Whole-image Norm. | 40.02% | 1794 | 356 | 13 | 197 | 7 | 13.13% | 3.79% | 0.89 | 0.15 | 0.94 | 0.97 |
| Foreground Norm. | 53.81% | 1896 | 410 | 22 | 164 | 10 | 15.23% | 4.17% | 0.88 | 0.16 | 0.93 | 0.96 |

MCF10A dataset

|  | Object-level metrics | | | | | | | | Pixel-level metrics | | | |
| --- | --- | --- | --- | --- | --- | --- | --- | --- | --- | --- | --- | --- |
| Normalization  method | % of overlapping cells separated | Correct  detections | Incorrect  detections | Splits | Merges | Catastrophes | FN rate | FP rate | Mean IoU | RMSE | F1 | Pixel accuracy |
| Whole-image Norm. | 84.96% | 3150 | 160 | 3 | 63 | 1 | 5.57% | 2.28% | 0.94 | 0.17 | 0.97 | 0.97 |
| Foreground Norm. | 89.78% | 3199 | 149 | 5 | 41 | 0 | 6.02% | 2.43% | 0.93 | 0.18 | 0.97 | 0.97 |

**Mask R-CNN:**

Kaggle fluorescent dataset

|  | Object-level metrics | | | | | | | | Pixel-level metrics | | | |
| --- | --- | --- | --- | --- | --- | --- | --- | --- | --- | --- | --- | --- |
| Normalization  method | % of overlapping cells separated | Correct  detections | Incorrect  detections | Splits | Merges | Catastrophes | FN rate | FP rate | Mean IoU | RMSE | F1 | Pixel accuracy |
| Whole-image Norm. | 80.04% | 1919 | 402 | 35 | 68 | 17 | 25.33% | 4.05% | 0.85 | 0.18 | 0.92 | 0.96 |
| Foreground Norm. | 81.49% | 1868 | 377 | 38 | 70 | 9 | 26.60% | 3.86% | 0.85 | 0.18 | 0.92 | 0.96 |

MCF10A dataset

|  | Object-level metrics | | | | | | | | Pixel-level metrics | | | |
| --- | --- | --- | --- | --- | --- | --- | --- | --- | --- | --- | --- | --- |
| Normalization  method | % of overlapping cells separated | Correct  detections | Incorrect  detections | Splits | Merges | Catastrophes | FN rate | FP rate | Mean IoU | RMSE | F1 | Pixel accuracy |
| Whole-image Norm. | 95.65% | 3233 | 198 | 9 | 15 | 2 | 4.77% | 4.62% | 0.91 | 0.21 | 0.95 | 0.95 |
| Foreground Norm. | 95.56% | 3226 | 168 | 3 | 19 | 2 | 6.37% | 3.71% | 0.91 | 0.22 | 0.95 | 0.95 |

Comparison of segmentation performance for NuSeT, U-Net, Mask R-CNN trained with whole-image normalization and foreground normalization. Foreground normalization consistently improves the segmentation performance on most object-level metrics for both NuSeT and U-Net.
