# Supplementary material for: NuSeT: A deep learning tool for reliably separating and analyzing crowded cells
Source: PLoS Comput Biol. 2020 Sep 14;16(9):e1008193. doi: 10.1371/journal.pcbi.1008193 (PMC7515182; doi:10.1371/journal.pcbi.1008193)
Supplement: S5 Table — (DOCX) [file pcbi.1008193.s005.docx]

**S5 Table. Memory footprint, training and inference speed comparison for different models.**

|  | Memory footprint (Training) | Memory footprint (Inference) | Training Speed  (seconds/Mega pixel) | Inference Speed  (seconds/Mega pixel) |
| --- | --- | --- | --- | --- |
| U-Net | 7653 MB | 7655 MB | 1.22 | 1.31 |
| Mask R-CNN | 7653 MB | 7655 MB | 0.81 | 4.73 |
| NuSeT | 7653 MB | 7655 MB | 1.31 | 1.98 |
