# Supplementary figures and images for: NuSeT: A deep learning tool for reliably separating and analyzing crowded cells
Source: PLoS Comput Biol. 2020 Sep 14;16(9):e1008193. doi: 10.1371/journal.pcbi.1008193 (PMC7515182; doi:10.1371/journal.pcbi.1008193)

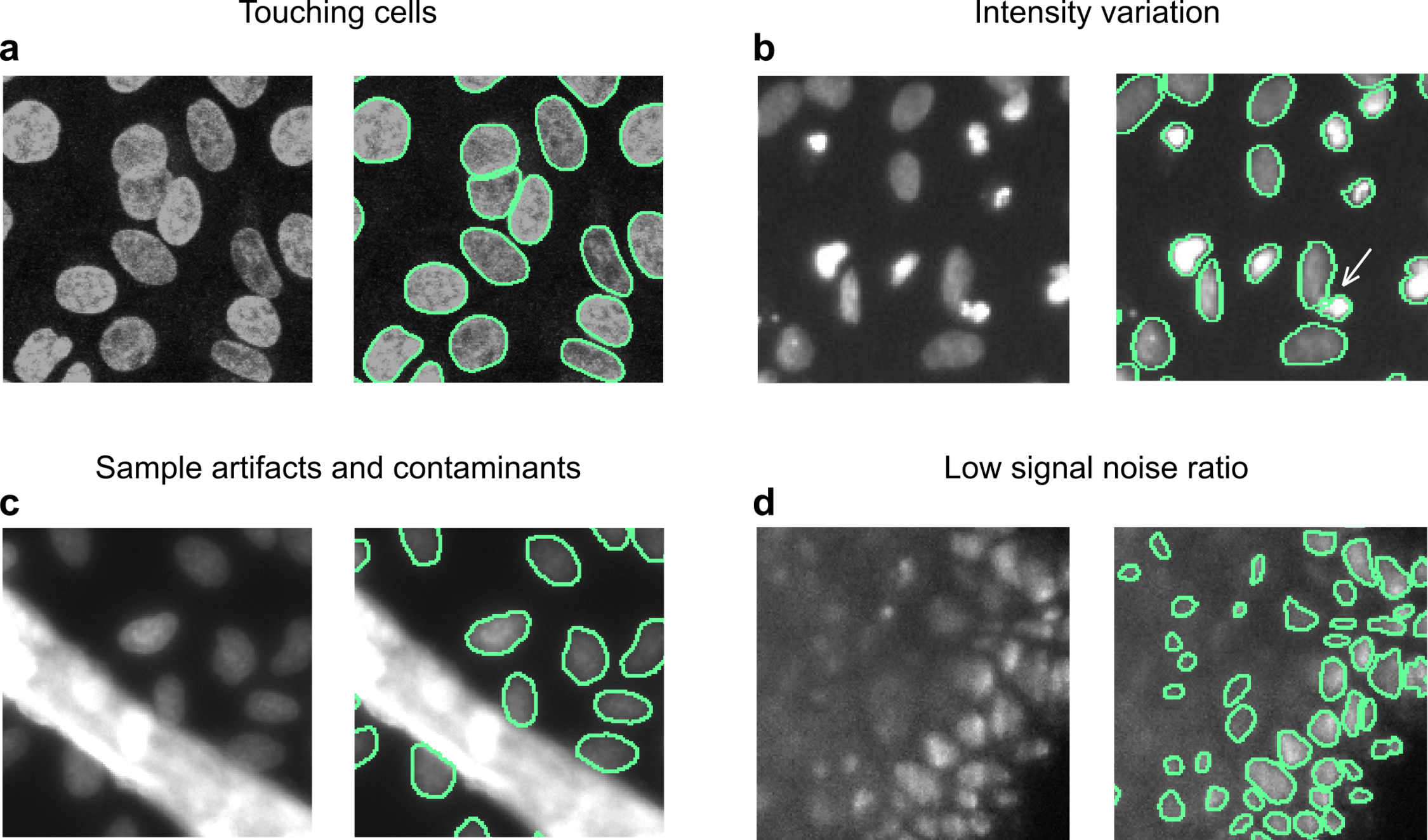

Supplement: S1 Fig — Some common factors that affect the quality of nuclei segmentation, are, touching cells (A), signal variation (B), sample preparation artifacts and contaminants (C), and low signal to noise ratio (D). Colored outlines represent the goals (ground truth) for segmentation tasks. (TIF) [file pcbi.1008193.s006.tif]

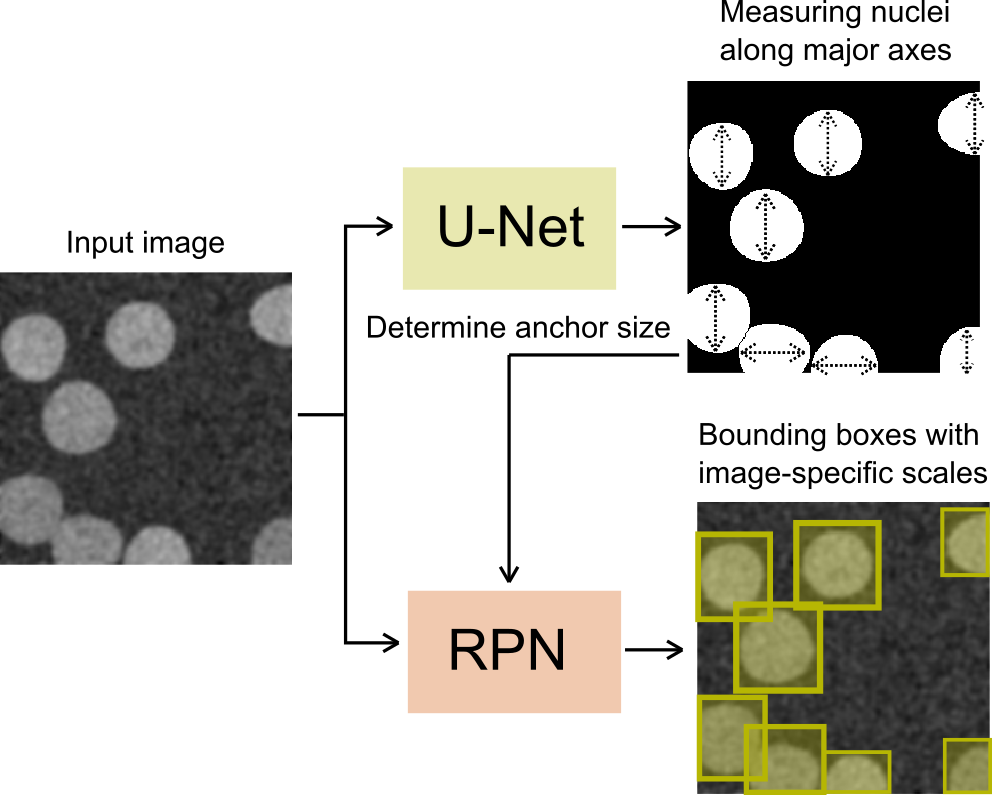

Supplement: S2 Fig — Historically RPN has used a set of rigid base sizes for all bounding boxes, which resulted in high detection error rate in the Kaggle dataset. We improved the RPN so that it applies different bounding box base sizes for different images. The base size is determined by the median of all nuclei sizes within the image. Nuclei sizes are defined by the maximum value between nuclei widths and heights. (TIF) [file pcbi.1008193.s007.tif]

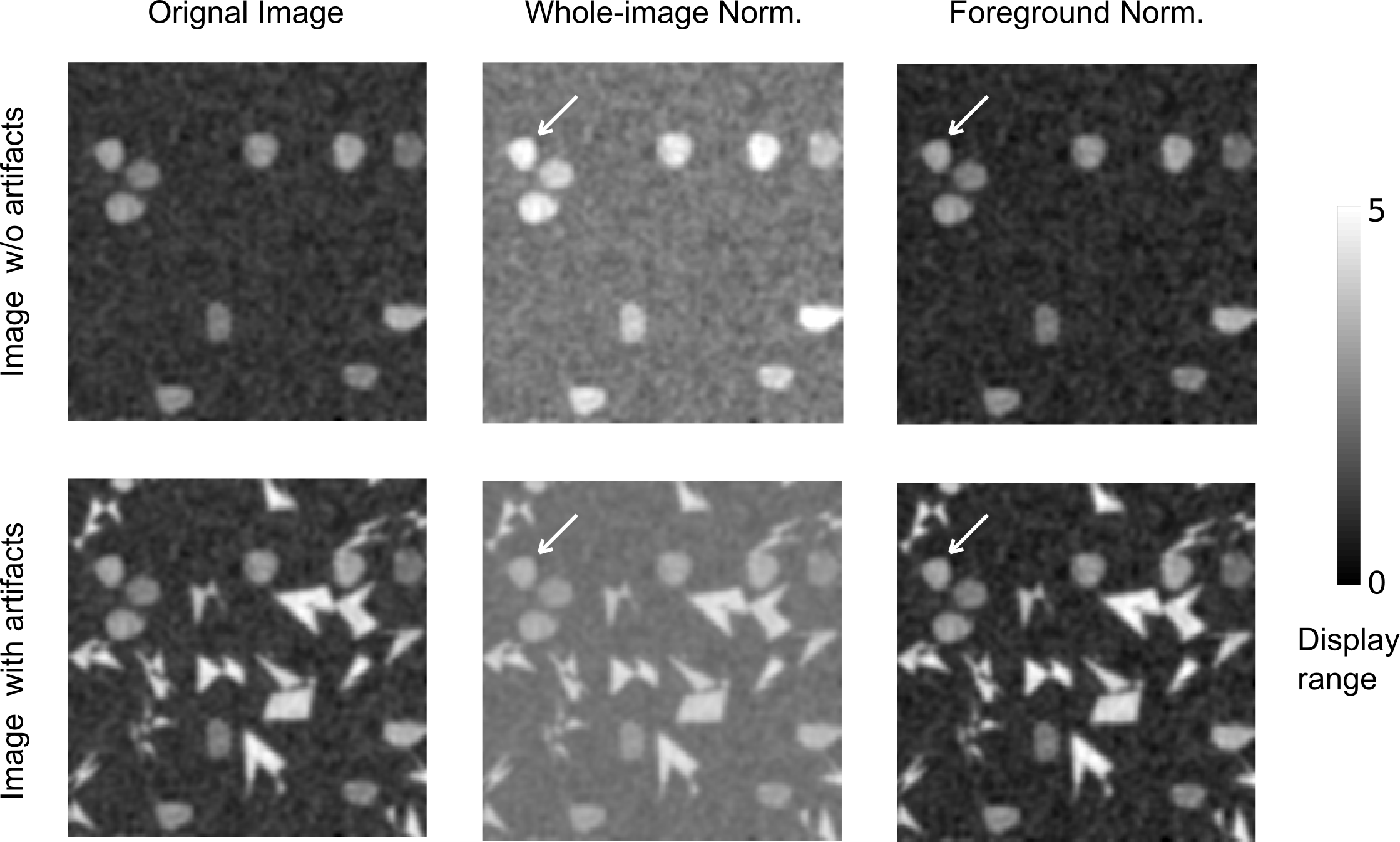

Supplement: S3 Fig — Normalizing samples with or without sample artifacts using different normalization methods show that images have more consistent nuclei signals after foreground normalization (highlighted by arrows). (TIF) [file pcbi.1008193.s008.tif]

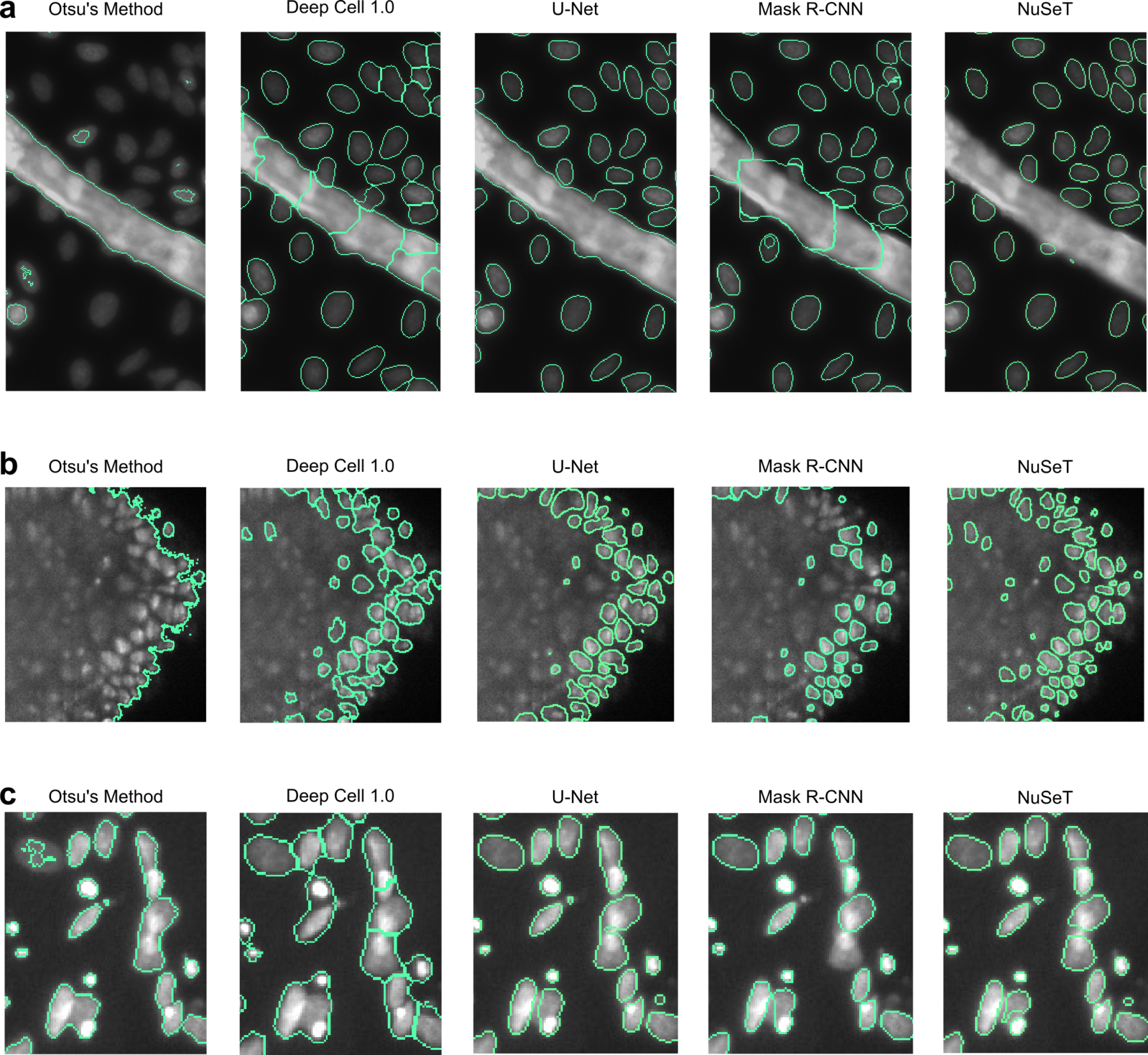

Supplement: S4 Fig — (TIF) [file pcbi.1008193.s009.tif]

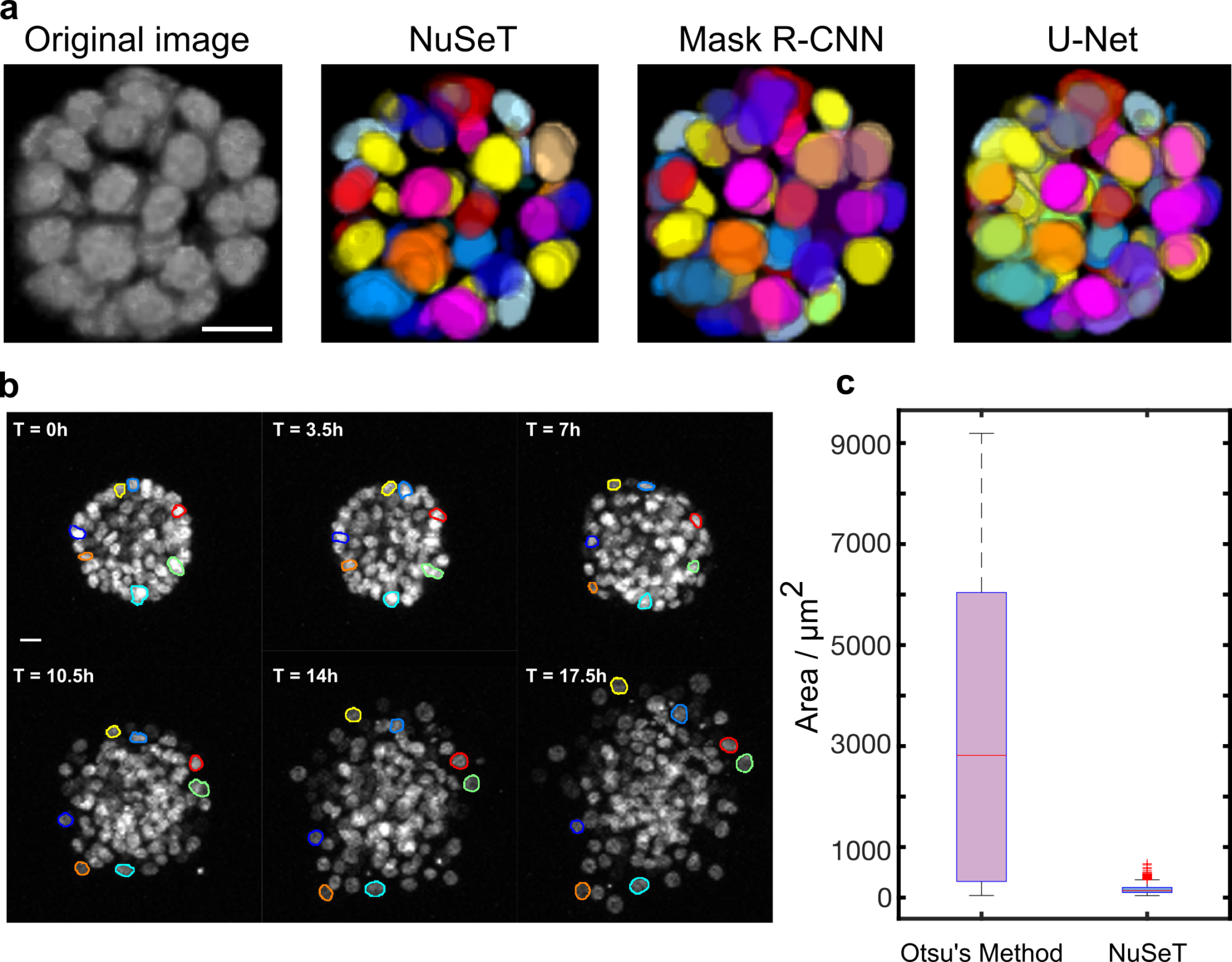

Supplement: S5 Fig — (A) Three-dimensional acini tracking with different deep-learning models. (B) Additional time-lapse tracking of selected nuclei. (C) Comparison of nuclei area distribution for Otsu’s method (median area: 2816.6 ± 2845.0 μm2) and NuSeT (median area: 138.7 ± 87.2 μm2). (TIF) [file pcbi.1008193.s010.tif]

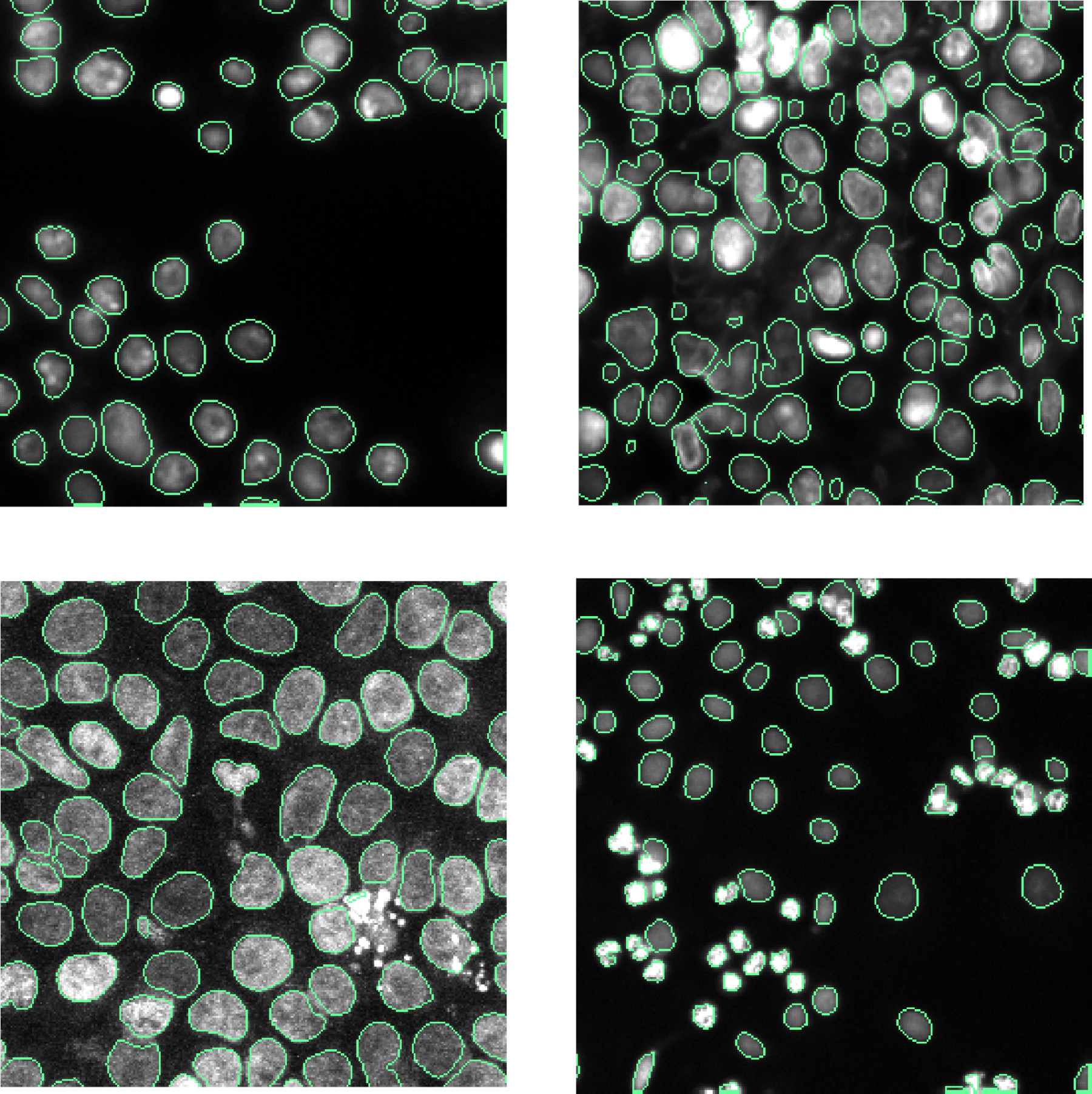

Supplement: S6 Fig — (TIF) [file pcbi.1008193.s011.tif]

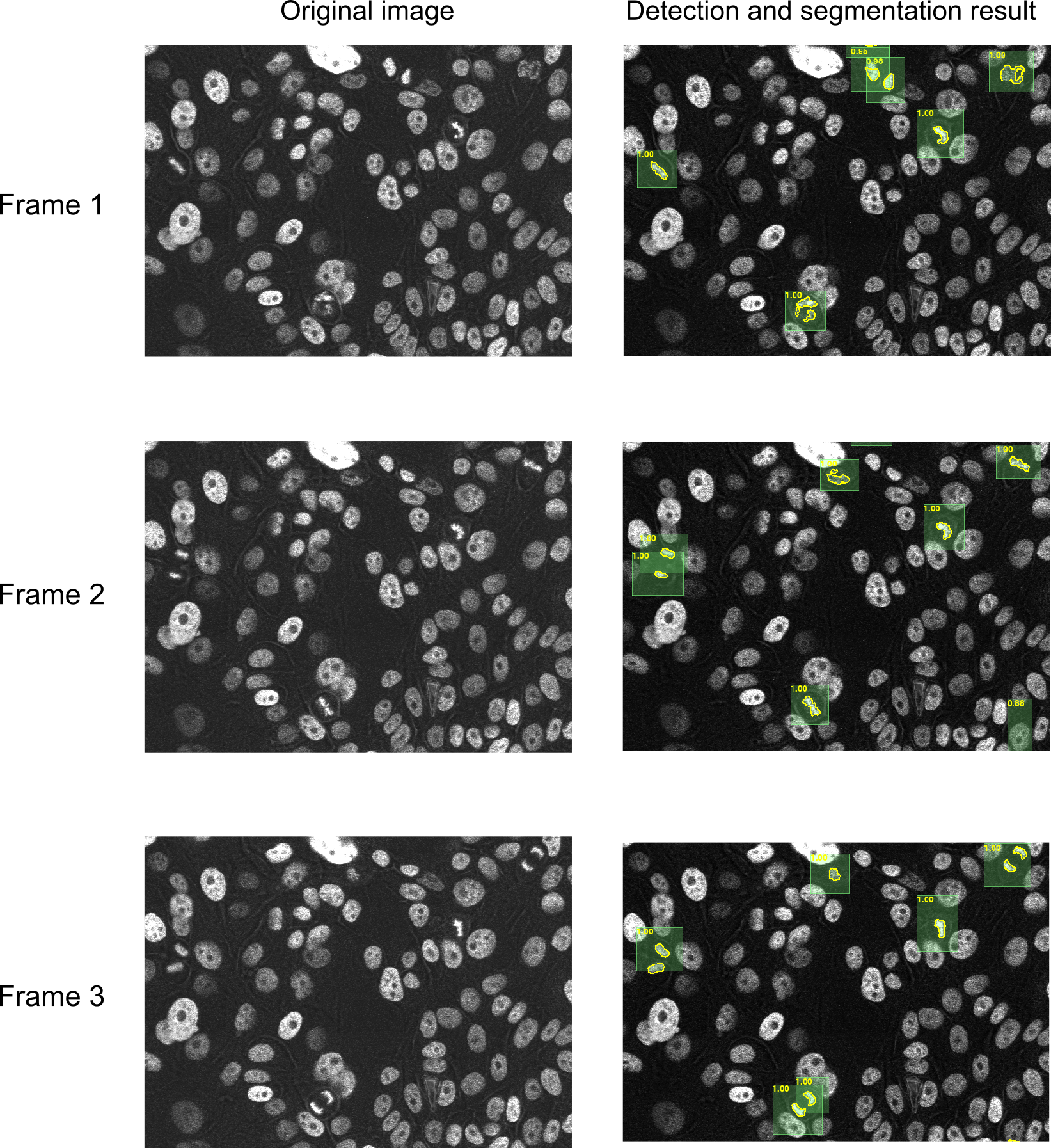

Supplement: S7 Fig — (TIF) [file pcbi.1008193.s012.tif]
