# Supplementary material for: NuSeT: A deep learning tool for reliably separating and analyzing crowded cells
Source: PLoS Comput Biol. 2020 Sep 14;16(9):e1008193. doi: 10.1371/journal.pcbi.1008193 (PMC7515182; doi:10.1371/journal.pcbi.1008193)
Supplement: S1 Text — (DOCX) [file pcbi.1008193.s013.docx]

**S1 Text. Supplementary notes about the NuSeT user interface (UI)**

NuSeT was built on python 3, please use python 3.5.x for the best compatibility:

https://www.python.org/downloads/

The following packages are needed for NuSeT to run:

1. Tensorflow (*pip install tensorflow / pip install tensorflow-gpu*)

2. PIL (*pip install Pillow*)

3. Numpy (*pip install numpy*)

4. Scikit-image (*pip install scikit-image*)

5. Tqdm (*pip install tqdm*)

Please download the NuSeT repository: <https://github.com/yanglf1121/NuSeT>.

To install all packages, simply run *python setup.py* in the root directory

After finishing installing packages, download 2 weight files from google drive: <https://drive.google.com/file/d/1fcs1F2lGPX0ejzEGPZ63YNF3AmUbdBcM/view?usp=sharing>

<https://drive.google.com/file/d/1hythQfvD6kbaUClAPY96nHcXB7RXVmBx/view?usp=sharing>

Move those files to Network/ folder. Then navigate to the root folder of this repo, in the command line run *python3 NuSeT.py.* The User Interface (UI) will pop up as below.


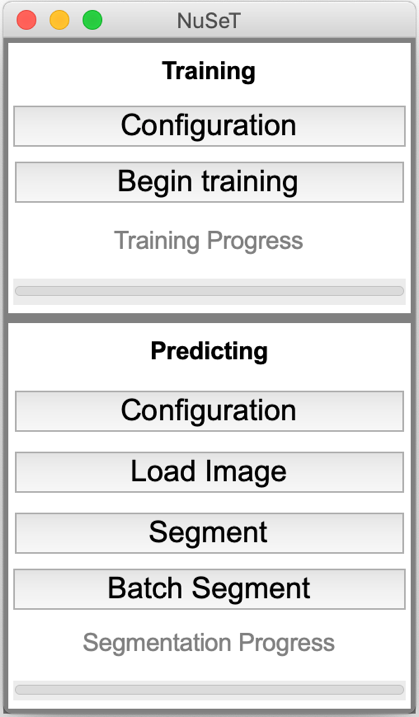


**Fig 1. The main menu of NuSeT software.**

This User Interface (UI) has been separately tested for Ubuntu (Linux), Mac and Windows, and passed all tests in three platforms. If Mac is used, the NuSeT UI will pop up as in Fig 1. There are two modules in the UI, namely **Training** and **Predicting**. The training module has two functions: **Configuration** allows you to choose basic training parameters, including the training models, number of training cycles (epochs), learning rates, and optimizers (Fig 2). Currently only Adam and Rmsprop optimizers are allowed, since they proved to work better in practice. Click the **Save** button when finished setting training parameters. Alternatively, user can choose the default parameters (NuSeT model, 35 epochs, learning rate 0.0001, Rmsprop) by simply ignoring this function.


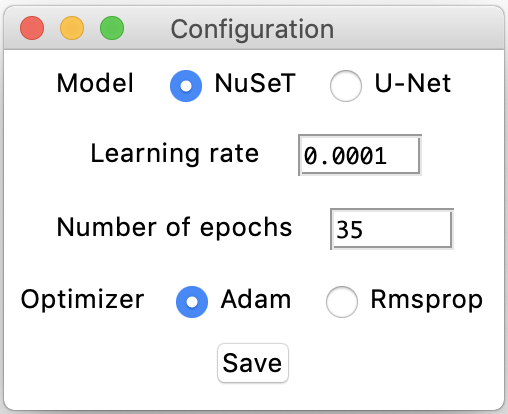


**Fig 2. The configuration page for NuSeT training module.**

Before the training, the training images and training labels should be stored in separate folders, and the name of the images and the corresponding training labels should be the same.

After closing the configuration window, click **Begin training**, and NuSeT will ask for the training image directory and training label directory. Upon choosing both directories, training will automatically start (Fig 3). The neural network will start with training a whole image normalization model, and then apply the whole image normalization results to train the foreground normalization model (NuSeT), user can also choose to train a U-Net model. This training step can take a long time.

­
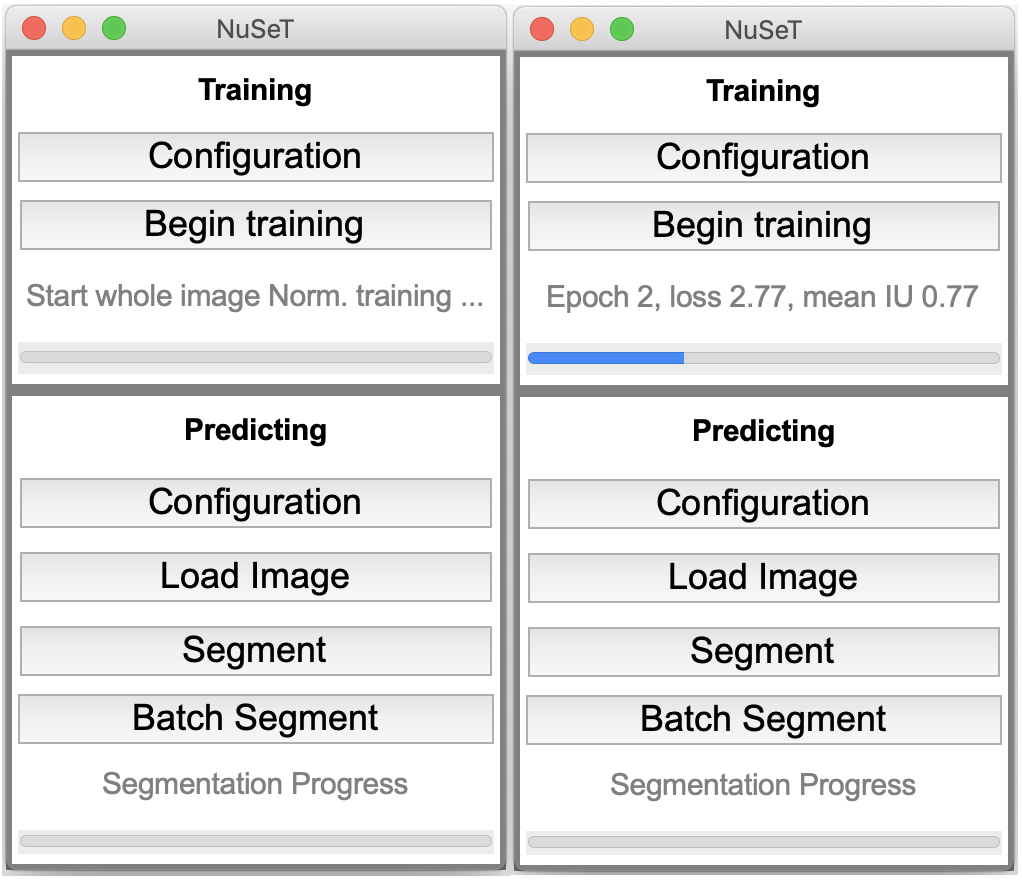


**Fig 3. Sample UI displays during the training process.**

Once the training is finished, the trained model will be saved in the Network/ folder, and then we are ready to move to the Predicting module.

Predicting module also allows you to choose several segmentation parameters by clicking the configuration button (Fig 4). First it asks the user whether they need the RPN-aided watershed transform. Then NuSeT asks the user to define the following parameters: Min detection score and Non-max-suppression (NMS) ratio, which are used to control the cell-detection confidence. Briefly, lower min detection score and higher NMS ratio allows more cells to be detected and separated, but the segmentation error will also increase accordingly. Post-processing will fill holes in binary masks and remove objects that are too small compared to a normal cell. Since NuSeT works better for moderate sized nuclei/cells, adjust the resize ratio of input image may lead to better performance. For optimal nuclei/cells sizes, please refer to our Github repository. Click **Save** to finish the parameter setup. Likewise, users can leave the default parameters (with watershed, Min detection score 0.85, NMS overlapping ratio 0.1, with post-processing and original image) by ignoring the configuration function.


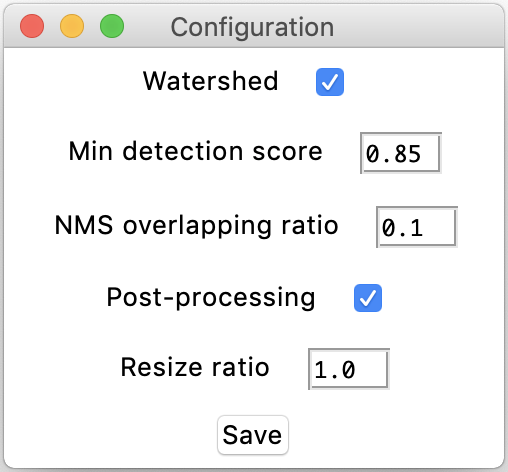


**Fig 4. The configuration page for NuSeT predicting module.**

We have provided a pre-trained model that was used in the paper to segment fluorescent nuclei, which was saved in the default Network/ folder, however user can also apply their trained model, which will automatically overwrite the original model when training is finished. NuSeT also allows user to visualize sample segmentation result for single image by clicking the **Load Image** button and **Segment** button (Fig 5).


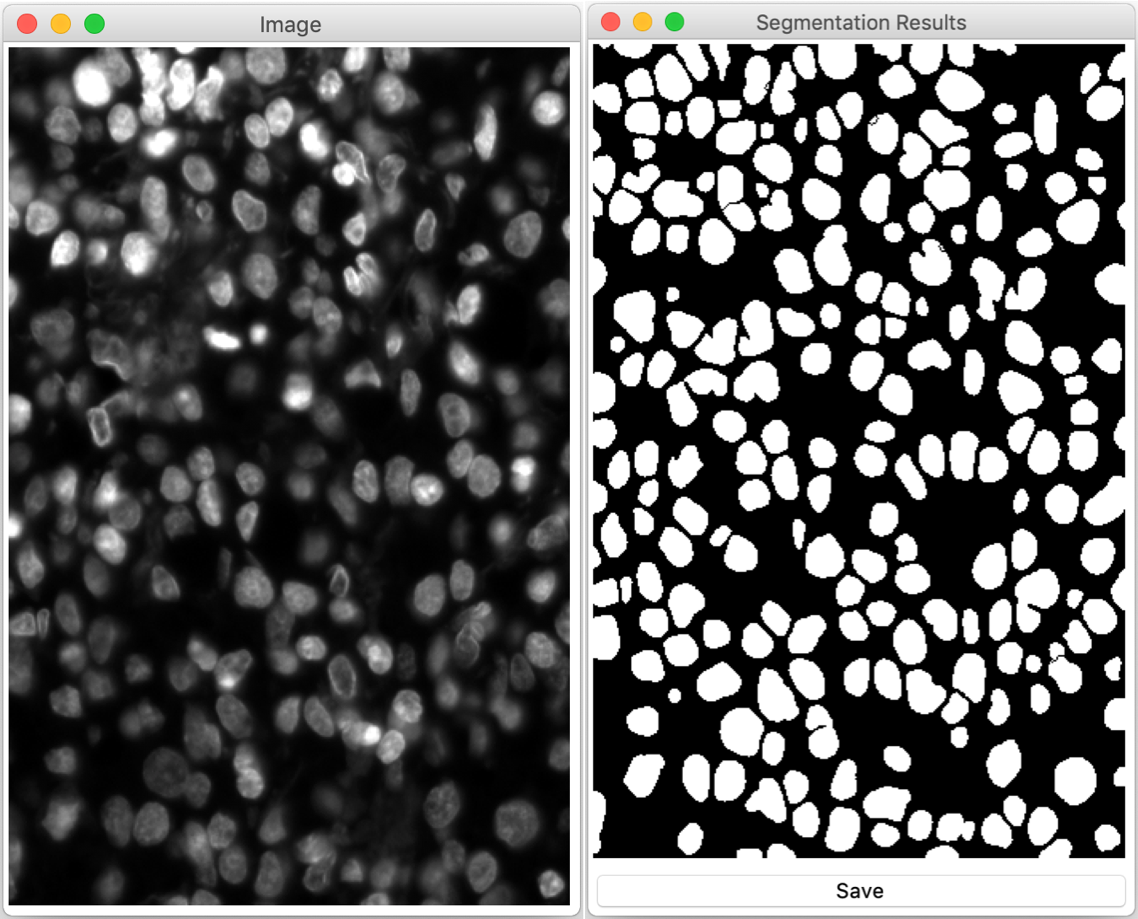


­­

**Fig 5. Example segmentation result using pre-trained NuSeT model.**

Once the segmentation results have been validated, NuSeT allows the user to segment all images within the given directory (**Batch Segment** button), and all segmentation results (binary masks) will be stored in the same directory as the images.
